# Exploring the function and effectiveness of knowledge brokers as facilitators of knowledge translation in health-related settings: a systematic review and thematic analysis

**DOI:** 10.1186/s13012-015-0351-9

**Published:** 2015-11-20

**Authors:** Catherine C. Bornbaum, Kathy Kornas, Leslea Peirson, Laura C. Rosella

**Affiliations:** Dalla Lana School of Public Health, University of Toronto, 155 College Street, 6th Floor, Toronto, ON M5T 3M7 Canada; Health & Rehabilitation Sciences, Western University, Elborn College, Room 2200, London, ON N6A 1H1 Canada; Public Health Ontario, Santé publique Ontario, 480 University Avenue, Suite 300, Toronto, ON M5G 1V2 Canada; McMaster Evidence Review and Synthesis Centre, School of Nursing, McMaster University Faculty of Health Sciences, 1280 Main St. W., Hamilton, ON L8S 4L8 Canada; Institute for Clinical Evaluative Sciences (ICES), G1 06, 2075 Bayview Avenue, Toronto, ON M4N 3M5 Canada

**Keywords:** Knowledge broker, Knowledge translation, Knowledge transfer, Linkage agent, Capacity builder, Knowledge manager, Evidence-based, Health, Evaluation, Systematic review

## Abstract

**Background:**

Knowledge brokers (KBs) work collaboratively with key stakeholders to facilitate the transfer and exchange of information in a given context. Currently, there is a perceived lack of evidence about the effectiveness of knowledge brokering and the factors that influence its success as a knowledge translation (KT) mechanism. Thus, the goal of this review was to systematically gather evidence regarding the nature of knowledge brokering in health-related settings and determine if KBs effectively contributed to KT in these settings.

**Methods:**

A systematic review was conducted using a search strategy designed by a health research librarian. Eight electronic databases (MEDLINE, Embase, PsycINFO, CINAHL, ERIC, Scopus, SocINDEX, and Health Business Elite) and relevant grey literature sources were searched using English language restrictions. Two reviewers independently screened the abstracts, reviewed full-text articles, extracted data, and performed quality assessments. Analysis included a confirmatory thematic approach. To be included, studies must have occurred in a health-related setting, reported on an actual application of knowledge brokering, and be available in English.

**Results:**

In total, 7935 records were located. Following removal of duplicates, 6936 abstracts were screened and 240 full-text articles were reviewed. Ultimately, 29 articles, representing 22 unique studies, were included in the thematic analysis. Qualitative (*n* = 18), quantitative (*n* = 1), and mixed methods (*n* = 6) designs were represented in addition to grey literature sources (*n* = 4). Findings indicated that KBs performed a diverse range of tasks across multiple health-related settings; results supported the KB role as a ‘knowledge manager’, ‘linkage agent’, and ‘capacity builder’. Our systematic review explored outcome data from a subset of studies (*n* = 8) for evidence of changes in knowledge, skills, and policies or practices related to knowledge brokering. Two studies met standards for acceptable methodological rigour; thus, findings were inconclusive regarding KB effectiveness.

**Conclusions:**

As knowledge managers, linkage agents, and capacity builders, KBs performed many and varied tasks to transfer and exchange information across health-related stakeholders, settings, and sectors. How effectively they fulfilled their role in facilitating KT processes is unclear; further rigourous research is required to answer this question and discern the potential impact of KBs on education, practice, and policy.

**Electronic supplementary material:**

The online version of this article (doi:10.1186/s13012-015-0351-9) contains supplementary material, which is available to authorized users.

## Background

Ensuring timely and optimal use of research evidence in health-related settings presents an ongoing challenge to practitioners and decision-makers [1]. Failure to optimize the use of research evidence may result in reduced quality of care [2], inefficient use of resources [3, 4], and poorer health outcomes for individuals and communities [5]. To mitigate the challenges associated with knowledge sharing between researchers, practitioners, and decision-makers [6], some knowledge translation (KT) experts have advocated for the use of an intermediary, known as a knowledge broker (KB) [7, 8].

KBs have been described as ‘knowledge managers’, ‘linkage agents’, and ‘capacity builders’ [8, 9]. Knowledge management tasks are related to the facilitation or management of the creation, translation, diffusion, and application of knowledge [8, 9]. Linkage and exchange activities focus on the development of positive relationships between knowledge creators (e.g. researchers) and knowledge users (e.g. decision-makers, clinicians) as a means to stimulate new information, collaborative knowledge exchange, and the use of evidence-informed approaches [8]. Capacity building activities aim to develop knowledge users’ understanding and skills [8], enable evidence-informed decision-making [10], and enhance capacity to access and apply knowledge [11]. Despite these distinct descriptions, in reality, KBs likely operate as an amalgam of these roles, depending on the goals of the KT initiative [12].

Essentially, KBs work collaboratively with stakeholders to facilitate the transfer and exchange of relevant information. They represent the human component of KT strategies as they work to facilitate interaction; develop mutual understanding of stakeholders’ goals and contexts; identify emerging areas of concern warranting attention; expedite the identification, evaluation, and translation of evidence into practice and/or policy; and facilitate the management of relevant knowledge [13, 14]. While KBs have operated in the private sector for years [8, 13], their adoption by the health sector has been rather limited until recently.

In 2003, the Canadian Health Services Research Foundation (CHSRF) developed a report on the theory and practice of knowledge brokering in Canada’s health system [13], which acknowledged the need for additional evidence to assess the efficacy of KB approaches and best practices. Others have echoed this recommendation [15–18]. While some have advocated for the use of KBs as a mechanism to facilitate KT [17, 19, 20], others suggest that the lack of evidence about how knowledge brokering works and its potential effectiveness limits the development and application of the KB role [8]. To address this gap, we sought to (1) identify and examine the activities and tasks which comprised the KB role in health-related settings and (2) assess whether KBs have effectively contributed to KT in health-related settings.

## Method

### Overview

We employed a systematic review and thematic analysis to synthesize and appraise diverse evidence related to knowledge brokering in health-related settings. Our thematic analysis [19] explored how KBs function in health-related settings. To assess whether KBs have effectively contributed to KT in health-related settings, we employed a systematic review, which permits an overall assessment of effectiveness through a comprehensive and reproducible search and assessment of existing literature [20]. Since the KB role may be influenced by myriad contextual factors [21], quantitative, qualitative, and mixed-method designs were assessed in addition to grey literature sources to elucidate the activities and tasks that comprised the KB role in health-related settings. While some have noted concerns regarding the feasibility and validity of synthesizing different research approaches [22], the objectives of this inquiry—aimed largely at exploring occupational processes and outcomes in diverse health settings—required a broader perspective than would be offered by limiting to a single research design.

### Search strategy

In collaboration with the research team, research librarians developed and implemented search strategies in eight electronic databases (MEDLINE, Embase, PsycINFO, CINAHL, ERIC, Scopus, SocINDEX, and Health Business Elite) using English language restrictions and covering all published work available up to November 2014 (Additional file 1). Websites of relevant KT networks (i.e. Canadian Foundation for Healthcare Improvement, National Collaborating Centre for Methods and Tools) and health-focused organizations (i.e. Canadian Institutes of Health Research, Canadian Public Health Information, Health Evidence, Ontario Public Health Units, World Health Organization (WHO), WHO’s ‘Brokering knowledge and Research Information to support the Development and Governance of health systems in Europe’ [BRIDGE] series) were searched in an effort to identify relevant grey literature (Additional file 2). Additionally, relevant journals and reference lists of included articles were reviewed.

### Study inclusion and exclusion criteria

To be included, studies must have reported on an actual application of knowledge brokering (i.e. theoretical assumptions about knowledge brokering were excluded). Studies must also have been available in English and occurred in a health-related setting, i.e. health-related contexts or environments including the following: healthcare practice (e.g. clinical, public health, rehabilitation, community-based health settings), health policy (e.g. interactions with health decision-makers at local, regional, provincial/state, federal or international levels), health education (e.g. interactions with health educators in clinical or academic settings), and healthcare administration (e.g. interactions with health system organizations). While studies were not excluded based on research design, when reviews were identified, we sought to locate the primary source document(s).

### Study selection

**Fig. 1:**
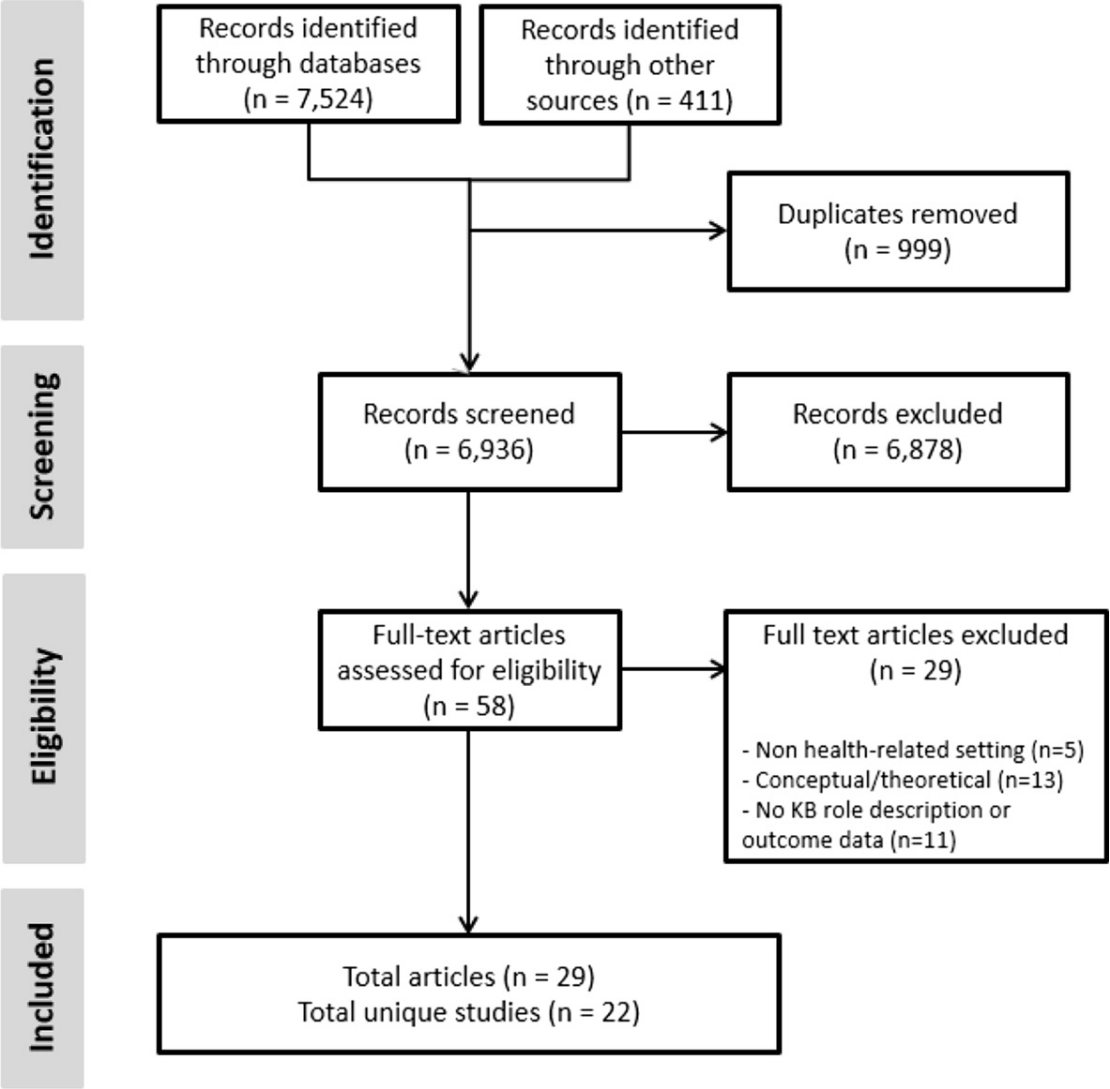
Flow diagram of process to identify eligible studies. Note: records identified through ‘other sources’ include grey literature, hand searching of relevant journals, and reference lists of manuscripts included in this review

Once search results were compiled and duplicates were removed, two reviewers independently screened the remaining records (i.e. titles and abstracts of articles or grey literature sources) for eligibility (Fig. 1). Subsequently, full-text articles and grey literature sources were independently assessed by two reviewers for alignment with inclusion criteria. Disagreements were resolved through discussion or third party adjudication. Multiple publications addressing the same KB initiative were combined into unique studies.

### Quality assessment

We assessed the methodological quality of all studies reporting outcomes related to the effectiveness of KBs (defined as changes in stakeholders’ knowledge, skills, policies, and/or practices [23]) using the Meta Quality Appraisal Tool (MetaQAT) [24]. MetaQAT combines enhanced principles of quality appraisal with the rigour of risk of bias assessment using select existing design-specific companion tools within a larger conceptual framework to guide their use in the context of broad health-related settings. Specifically, MetaQAT provides a set of rigourous methodological guidelines to synthesize diverse types of evidence (e.g. quantitative, qualitative, mixed methods, grey literature). It consists of a four-step critical appraisal framework which assesses relevancy, validity, reliability, and applicability. It also contains research design-specific modules for quantitative (e.g. PRISMA, CONSORT, TREND, AGREE, CASP), qualitative (e.g. McMaster critical review form: qualitative studies (version 2)), and mixed methods (e.g. Evaluation Tool for Mixed Methods Studies) research design appraisal; thus, demonstrating broad applicability across study designs, which is a fundamental requirement of a multi-modal quality appraisal tool [25]. Importantly, MetaQAT has undergone a transparent development and validation process [24]. Appropriate studies were appraised by two independent reviewers with disagreements resolved by third party consultation.

### Data extraction

Data were extracted using standard forms developed for this protocol and included the following: setting(s), purpose of the initiative, duration of the KB initiative, level of KB’s experience (e.g. novice, experienced), KB’s position status (e.g. full-time, part-time), KB approach (e.g. independent, team-based), and whether the KB role was embedded in or external to the organization(s) (i.e. internal employee or externally contracted). We also explored strategies used by KBs to promote KT (e.g. in-person meetings, teleconferences). To assess the effectiveness of KBs at facilitating KT, data pertaining to changes in knowledge, skills, policies, and practices were also extracted [23]. Extracted data were reviewed and approved by both reviewers; disagreements were resolved by discussion.

### Data analysis and synthesis

#### Thematic analysis

In line with our first objective to improve conceptualization of the KB role in health-related settings, we conducted a confirmatory thematic analysis [19] to assess the operationalization of the KB role according to the domains described by Ward et al. [8] and Oldham and McLean [9] (i.e. knowledge management, linkage and exchange, and capacity building). Extracted data were analyzed using NVivo9 [26]. A deductive approach was employed [27], which involved a priori construction of a preliminary coding manual structured according to the sensitizing concepts (knowledge management, linkage and exchange, and capacity building). This approach was complemented by inductive coding to identify emergent themes. Extracted data were initially synthesized by one reviewer. The draft synthesis was reviewed by a second reviewer and iteratively adapted until agreement on appropriateness of themes and subthemes was achieved. Any disagreements were resolved through discussion.

#### Assessment of effectiveness

To address our second objective to determine whether KBs contributed to effective KT in health-related settings, we explored outcome data for evidence of changes in knowledge, skills, policies, or practices. Our approach was adapted from the work of Kujbida and Stratton [23] who measured changes in attitude, knowledge, and practice (among other factors) to assess the effectiveness of KT strategies. For studies presented in more than one publication, all relevant articles were analyzed collectively to ensure examination of relevant contextual factors. Our review sought to answer the following research question: Are knowledge brokers an effective mechanism to facilitate KT relative to reported changes in knowledge, skill, policies, and/or programmes in health-related settings among KT participants?

## Results

Twenty-nine articles, representing 22 unique studies, met our inclusion criteria and were included in the review (Fig. 1). Qualitative (*n* = 18), quantitative (*n* = 1), and mixed methods (*n* = 6) research designs were represented in addition to grey literature sources (*n* = 4). Studies were heterogeneous relative to health-related settings, length of KB initiative, KB approach, KB position status, and whether the KB was internal or external to the participating organization(s). Descriptive characteristics of the 22 studies are presented in Additional file 3.

### Activities and tasks of KBs

Findings indicate that KBs in health-related settings performed a diverse range of tasks across the three domains proposed by Oldham and McLean [9] and Ward et al. [8], thus supporting the KB role as a knowledge manager, linkage agent, and capacity builder. Moreover, findings suggested that KB activities often overlapped these theoretical constructs. Our thematic analysis generated ten main KB activities. Below, we introduce each of these activities and elaborate on their associated tasks. Table 1 provides a list of the KB tasks identified in the studies and shows how they are connected to the general domains of activity.

**Table 1:**
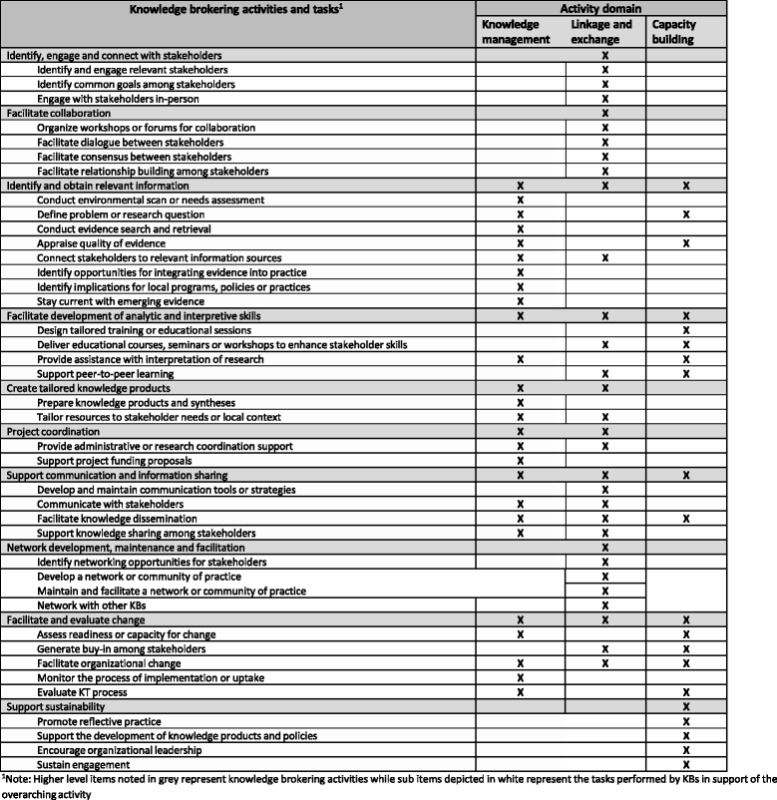
Classification of knowledge brokering tasks according to activity domains

#### Identify, engage, and connect stakeholders

KBs worked to identify and connect with stakeholders with relevant expertise [28, 29], and key individuals or organizations who were working on similar problems [15, 30] or in similar areas of research [29]. Specifically, this task involved finding the ‘right’ people [15, 31] or organizations to support the KT objectives and then garnering their participation [32–34] through telephone, electronic, or in-person contact [15]. Maintaining a physical presence among stakeholders was also noted to be useful [35]. To support stakeholder engagement, KBs identified common goals among stakeholders by helping to clarify their needs [30, 36, 37], identifying mutually beneficial opportunities [17], and bringing together individuals with common interests and relevant expertise to address the issue [15, 29]. Specifically, KBs engaged in-person through site visits to stakeholders’ organizations [21] and meetings [29, 38] that included both one-on-one [34] and larger group [29, 34, 39] discussions.

#### Facilitate collaboration

KBs worked to facilitate collaboration by organizing group forums such as workshops [17, 31, 40], journal clubs [41], online forums [15, 41], and multi-sector advisory committee meetings [41]. To promote collaboration, KBs facilitated dialogue between stakeholders by establishing communication channels [31], creating a ‘safe’ forum to share research activities [31], facilitating group discussions or problem-solving sessions [15, 28, 29, 39, 40], clearing up misunderstandings [42], leading focus groups [15], and chairing teleconferences [15]. KBs facilitated consensus by assisting stakeholders to clarify their needs and expectations [17, 36], helping stakeholders to understand each other’s standards of methodological rigour [31], and negotiating shared project objectives [17, 28, 31, 36], deliverables [31], and outcomes [31].

In addition, KBs facilitated relationship building among stakeholders [15, 29, 31, 38, 43, 44] by helping to negotiate the terms of partnerships [15, 31], encouraging teamwork [15, 44], and facilitating interactions [43].

#### Identify and obtain relevant information

KBs conducted environmental scans [15, 21, 32, 33, 45] and needs assessments [15, 21, 39, 46] to identify local needs [28, 37, 38, 47], gauge the scope of the project [28, 36], determine available resources [15], and analyze organizational capacity [32, 33]. They also worked with stakeholders to define problems or research questions by translating clinical/management questions [37, 47] or policy gaps into operationalizable research questions [17, 28, 30, 31, 36], by helping stakeholders to formulate research priorities based on policy concerns [17, 30], and by working with practitioners to identify practice areas where research findings would be useful [37].

After defining the research question, KBs conducted searches to identify and gather useful information [17, 37, 39, 45, 46, 48], which was sometimes managed through reference software [38]. KBs then appraised evidence quality by assessing its relevance, credibility, and usefulness [43]; at times, they also built stakeholder capacity to interpret [21] and critically appraise the evidence [37]. Following appraisal, KBs connected stakeholders to the relevant information sources either directly [15, 35, 49] or through collaboration with library support staff [38] or networks [35]. KBs also identified opportunities to integrate evidence into practice [39, 42] and determined implications for local programmes, policies, and practices [21, 37] by providing knowledge about frontline practices [50] and conducting health system-specific analyses [30]. Lastly, KBs made an effort to stay current with emerging evidence in KT methods and the specific content area(s) by maintaining subscriptions to listservs [21, 38, 46], e-table of content alerts from relevant journals or really simple syndication (RSS) feeds [38, 46], bookmarking relevant websites [21], reading journal articles [39], cataloguing resources that could be useful [15], and using available training materials [39].

#### Facilitate development of analytic and interpretive skills

To facilitate the development of stakeholders’ analytic and interpretive skills, KBs designed [15, 33, 40, 47] and delivered [15, 28, 35, 37, 40, 48, 49] educational initiatives for policy makers [28, 31–33] and clinicians [21, 35, 37, 39, 44–46], which included workshops [21, 28, 32–34, 37–39, 48], seminars [34, 37, 39], webinars [21], courses [34], public lecture series [34], informal mentorship [48, 49], and public meetings with international experts [34]. These sessions aimed to enhance evidence-informed decision-making [28, 31–33, 50] and practice [37, 39, 44, 45], develop critical appraisal skills [21, 48], increase understanding of KT theory and processes [15], and enhance technical skills or subject-specific knowledge [15, 32, 34, 39, 45]. KBs were also noted to provide ongoing learning opportunities [44], to teach in clinical settings [45] and role-model desired behaviours (e.g. using evidence to inform decisions) [45]. KBs also assisted with the interpretation of research [35, 37, 46] and supported peer-to-peer learning (e.g. stakeholder-led education sessions) [15, 44].

#### Create tailored knowledge products

KBs prepared tailored knowledge products and syntheses for stakeholders by summarizing evidence [28, 35, 37, 47, 49], translating relevant findings to the local context [17, 21, 37, 41, 44, 45], and writing or supporting the preparation of tailored knowledge products [17, 21, 30, 37, 49, 51] (e.g. resource binders [39], reports [30, 34], policy briefs [28, 32, 33], logic models [49], clinical reasoning flowcharts [35], patient education materials [35], journal article summaries [35], blogs [35], presentations [33], fact sheets [33], newsletters [15, 35], websites [37, 39], and peer-reviewed manuscripts [15]). KBs ensured that knowledge products were concise [28, 37], relevant to stakeholders’ needs [17, 28, 51], and presented in an accessible format [51]; the importance of maintaining transparency throughout the process was also noted [37].

To ensure knowledge products were relevant to stakeholder needs, KBs worked directly with stakeholders [30] to synthesize research findings with professional expertise [45]. KBs tailored evidence by evaluating, interpreting, and distilling information for different audiences [45] to determine what the main messages would mean for different stakeholders in their specific contexts [17, 49]; for instance, in one study, KBs translated patient safety recommendations into department procedures and provided staff with examples of how policies would translate into their local practice context [50].

#### Project coordination

KBs were often responsible for project coordination tasks such as developing and maintaining contact and distribution lists [15, 46], e-mail filing [21], planning and facilitating meetings and events [15], developing and updating websites [15, 37], managing web-based tools [15], liaising with information technology personnel [39], and maintaining a log to track stakeholder-related activities [21]. KBs also supported grant applications by conducting reviews [34] and drafting funding proposals [31].

#### Support communication and information sharing

To support information sharing, KBs established communication channels [29, 31] and initiated [46] and coordinated ongoing communication [15, 28, 35, 48] with stakeholders to provide professional updates though emails, briefings, and other forms of communications [35, 48–50]. To facilitate knowledge dissemination, KBs prepared research syntheses and facilitated access to evidence [17, 35] through websites and other forums [28, 30, 34, 35], provided summaries to practitioners making service-level decisions [37, 48], advocated effective policy briefs [17], presented findings to decision-makers [34, 49], and supported stakeholders in presenting policy briefs to high level officials to gain endorsement and implementation of the policy [33]. KBs also supported knowledge sharing by harnessing members’ expertise and sharing it with others [29], facilitating inter-organizational communication [35], and promoting internal knowledge sharing through team e-mail distributions and meetings with team members and management [21, 38].

#### Network development, maintenance, and facilitation

To support the linkage and exchange of information, KBs developed, maintained, and facilitated networks and communities of practice (CoP) for both stakeholder groups and themselves. KBs identified networking opportunities [21, 35] by connecting with professional groups [15, 47] and researchers [34], identifying individuals who could benefit from a CoP [15], and actively recruiting individuals and organizations [32] who were interested in similar issues [15]. KBs fostered the development of networks or CoPs [15, 21, 35, 38, 42, 46] by organizing joint forums for stakeholders [17, 49] and developing processes, policies, and reporting structures for the network [15]. Once the networks were established, KBs maintained network operations by developing strategic plans, facilitating information sharing, promoting and publicizing the network, supporting membership growth [15], and fostering relationships with researchers [30], academics [30, 34], and decision-makers across diverse sectors [29, 30, 34]. At times, KBs also networked directly with other KBs [15, 39, 52].

#### Facilitate and evaluate change

To evaluate readiness for change, KBs conducted needs assessments [15, 32, 38] and used evidence to generate stakeholder buy-in for the need for change [45]. KBs facilitated organizational change by developing change management strategies [15]; cultivating receptivity among stakeholders [15, 49]; encouraging decision-makers to act as role models (e.g. requiring evidence to support recommendations) [38]; and by leading the development and implementation of evidence-based guidelines [45], interventions [43], and programme plans [46]. Throughout these organizational changes, KBs monitored the impact of the changes on policies and key indicators [17, 35]. They also conducted ongoing evaluations throughout the process [35, 47] in an effort to ensure stakeholders used relevant evidence [45], that resources were responsive to stakeholder concerns [35], and to learn from the knowledge exchange process as a whole [43].

#### Support sustainability

To support sustainability of desired KT outcomes, KBs focused on building capacity and fostering self-reliance among stakeholders. For instance, they promoted reflective practice [35, 38] among stakeholders to increase awareness of self-practices related to evidence use. KBs also supported stakeholders to develop evidence-informed policies [31] and knowledge products including policy briefs [31–33], reports [30, 50], and books [48]. At times, KBs had a role in anticipating and stimulating the broader health agenda [30] to facilitate sustainability of stakeholder priorities. KBs also worked to sustain stakeholder engagement [29, 43] by advocating for dedicated staff time for KT activities [48], and by encouraging senior staff and decision-makers to include components of evidence-informed decision-making [21] in performance appraisals and staff professional development plans [21, 38].

### Effectiveness of KBs

Our second objective was to assess whether KBs have effectively facilitated KT in health-related settings. Accordingly, we explored outcome data from a subset of studies (*n* = 8) that reported evidence of changes in knowledge (*n* = 5), skills (*n* = 2), and policies or practices (*n* = 6) related to their KB strategies [17, 21, 32, 36, 38, 39, 42–44, 46, 48, 49, 51, 52]. Following assessment of methodological quality [24], two studies (i.e. Russell et al. [39, 44, 52] and Dobbins et al. [21, 38, 46, 49]) met standards for acceptable methodological rigour. One study reported a positive effect of the KB strategy on stakeholders’ knowledge and practices [39, 44, 52], while the other did not identify a statistically significant effect on stakeholders’ practices [21, 38, 46, 49]. Owing to the conflicted findings and limited methodological quality of other existing evidence, findings are inconclusive regarding the effectiveness of KBs in health-related settings. A summary of quality appraisal findings is presented in Additional file 4, while the specific changes in knowledge, skills, and policies or practices related to the KB initiatives are presented below.

#### Change in knowledge

Ward and colleagues [43] explored the nature of KB-facilitated knowledge exchange across three service delivery groups in mental health settings. Following the KB intervention, authors reported that one participant team broadened the scope of what they valued as ‘knowledge’ to include policy, service literature, and experiences of other service delivery teams. Additionally, Lyons and colleagues [42] reported on the Atlantic Stroke Care group’s experience with knowledge brokering to foster decision-makers’ uptake of best practices in integrated stroke care. Despite the project still being in progress, the authors reported that the KB initiative increased decision-makers’ knowledge of best practices for stroke care and researchers’ understanding of contextual factors. Waqa et al. [32] conducted KB-led workshops on evidence-informed policy brief development where all participants described increased knowledge regarding strategies to optimize the development of evidence-informed policy briefs (e.g. how and where to source evidence). In addition, Yost and colleagues [48, 49] evaluated the effectiveness of tailored KB strategies at three public health departments. They aimed to enhance capacity for evidence-informed decision-making through a series of site-specific strategies including one-on-one consultations, small group meetings, workshops, and presentations. They found that participants who worked closely with the KB demonstrated a statistically significant change in knowledge [49]. However, owing to methodological limitations, we cannot conclude that the KB interventions performed by Ward et al. [43], Lyons et al. [42], Waqa et al. [32], and Yost et al. [48, 49] were responsible for the reported changes to participants’ knowledge (Additional file 4).

Russell and colleagues [39, 44, 52] evaluated the impact of a 6-month KB intervention on changes in physiotherapists’ knowledge of four clinical assessment tools. Participants completed self-report questionnaires to assess their knowledge prior to the KB intervention, immediately following the intervention and again at 6 and 12 months post-intervention. Data revealed participants’ knowledge of all measurement tools significantly increased following the intervention and was sustained 1 year later, suggesting an effective KB approach. No significant methodological concerns were identified.

#### Change in skills

Waqa et al. [33] reported that their participants developed evidence-informed policymaking skills through a series of KB-led training workshops; they cited participants’ perceptions [32] and the production and presentation of 20 policy briefs by their participants to high-level officials [33] as evidence of this skill development. In addition, Yost and colleagues [48, 49] evaluated the effectiveness of tailored KB strategies to enhance capacity for evidence-informed decision-making and found that participants who worked closely with the KB demonstrated a change in evidence-informed decision-making skills [49]. However, owing to methodological limitations, we cannot conclude that the KB interventions performed by Waqa et al. [32] and Yost and colleagues [48, 49] were responsible for the reported changes to participants’ skills (Additional file 4).

#### Change in policies or practice

van Kammen et al. [51] described how a KB organization, The Netherlands Organisation for Research and Development, generated a report that resulted in policy revisions to the definition of in vitro fertilization treatment by the Dutch Society of Obstetrics and Gynecology. Additionally, Campbell et al. [36] reported ‘direct impacts on policy or practice’ (p. 104) as a result of their KB initiative, which described ‘evidence check’, an approach to providing policy makers with rapid reviews of evidence.

Also, Waqa et al. [32] performed a series of KB-led workshops on developing evidence-informed policy briefs and reported that policies to promote a healthy work environment were developed by three of the six participant organizations. Additionally, using tailored KB strategies, Yost and colleagues [48, 49] found that participants who worked closely with the KB demonstrated an increase in evidence-informed decision-making [49]. Unfortunately, owing to methodological limitations, we cannot conclude that the KB interventions performed by van Kammen et al. [51], Campbell et al. [36], Waqa et al. [32], and Yost and colleagues [48, 49] were responsible for the reported changes in policies and practices (Additional file 4).

Changes in practice were also reported by Russell and colleagues [39, 44, 52] who evaluated the impact of their KB intervention on changes in physiotherapists’ use of four clinical assessment tools. Participants self-reported their tool use via questionnaires delivered prior to the KB intervention, immediately following the intervention and again at 6 and 12 months post-intervention. With the exception of one tool, reported use of the tools in practice increased and the effect remained 1 year later suggesting an effective KB strategy. No significant methodological concerns were identified.

Dobbins et al. [21, 38, 46, 49] performed a randomized controlled trial to evaluate the impact of three KT strategies that aimed to incorporate research evidence into public health programmes and policies. The interventions focused on promoting healthy body weights in children and varied in intensity (i.e. access to a web-based repository of systematic reviews (least intensive); tailored, targeted messages plus access to the website (moderate intensity); KB support plus tailored, targeted messages and website access (most intensive)). Findings indicated that the KB strategy was not effective in promoting evidence-informed decision-making, although the authors noted a possible trend towards a positive effect when organizational research culture was low. Notably, high participant turnover and insufficient exposure to the intervention among health department staff may have contributed to the lack of observed effect of the KB intervention. While no significant methodological concerns were identified, the authors acknowledged challenges in applying an empirical research design to evaluate the effectiveness of KT strategies.

## Discussion

As the human component of KT, the KB role is based on the premise that interpersonal contact enhances the likelihood of behaviour change [53]. To date, evidence related to the role and effectiveness of KBs has been primarily anecdotal or theoretical in nature. However, given that KBs represent a costly and intensive KT strategy, it is important to both understand how they function and to establish rigourous evidence of their effect before widespread use is encouraged [49]. To our knowledge, the studies included in this review represent the current breadth of evidence exploring the functions and effectiveness of KBs in health-related settings. Despite the broad scope of our inquiry, there was a paucity of data related to the effectiveness of KBs. Nevertheless, a number of key findings were identified.

### Conceptualizing how KBs operate in practice

Given that there is currently no standard job description or widely accepted list of qualifications for KBs [49], this review sought to advance theoretical notions about knowledge brokering through a deeper understanding of the actual functions performed by KBs, which may inform KT-focused education and practice for current and future KBs. Over the past decade, KBs have operated widely across diverse, international health-related settings [17, 21, 32, 33, 36, 38, 39, 42–44, 46, 48, 49, 51, 52]. Despite heterogeneity in the settings, interventions, and role descriptions, we found that the activities and tasks which comprised these roles corresponded to the characterization of KBs as knowledge managers, linkage agents and capacity builders [8, 9]. Further, our findings revealed significant overlap between each of these role descriptions, confirming that KBs operated as an amalgam of the knowledge manager, linkage agent and capacity builder roles, depending on the scope and objectives of the KT initiative.

Despite our efforts to characterize existing KB activities and tasks, this description does not represent a comprehensive taxonomy of the role. A challenge to compiling a complete taxonomy of KB activities is that specific brokering activities are often difficult to standardize or define because the role requires flexibility and responsiveness to a stakeholder’s context and needs, both anticipated and emergent [52]. Moreover, while not captured in this review, the personal attributes [13, 16, 53] of a KB may also play an important role in how they operate in practice, thus introducing another dimension of measurement challenges. Ultimately, many of the functions and activities of a KB may emerge iteratively or be influenced by the needs of stakeholders and the attributes of the broker; so discerning the boundaries between these nuanced contextual factors poses a significant challenge to both conceptualizing the KB role and assessing the effectiveness of the broker.

### Effectiveness of KBs

In assessing the effectiveness of KBs in practice, we explored reported changes in knowledge, skills, policies, and practices related to the KB interventions. Following critical appraisal, two studies were found to be methodologically rigourous [21, 38, 39, 44, 46, 49, 52] but yielded conflicting results regarding KB effectiveness. Dobbins and colleagues [21, 38, 46, 49] reported that support for their KB strategy was detected only in those public health departments with a low organizational research culture, while Russell and colleagues [39, 44, 52] found that a strong research culture significantly predicted awareness and use of one of the four tools they assessed. Thus, the role of organizational context (e.g. readiness for change, organizational research culture) may warrant consideration when preparing a KB intervention; however, more research into this relationship is required.

Additionally, given that Dobbins’ intervention [21, 38, 46, 49] sought to support the incorporation of research evidence into public health policies and programmes, it is worthwhile to note that the KBs were not situated in the participating public health departments, and instead acted as an external resource to the participant sites. In contrast, Russell and colleagues’ [39, 44, 52] intervention focused on supporting the awareness and use of evidence-based assessment tools by physiotherapists via KBs who were embedded in the clinical sites and thus acted as an internal resource to the physiotherapist participants. Accordingly, the nature of the KB role (i.e. internal or external to the organization) and physical location of the broker may be important factors to consider when designing a KB intervention.

While the remaining six studies [17, 32, 33, 36, 42, 43, 48, 49, 51] reporting effectiveness data did not meet this review’s standards for methodological rigour, meaningful information about how KBs operate in practice can still be gleaned from these reports and the additional 14 studies that did not report outcome level data. In fact, every study included less tangible or more ‘subtle’ impacts of knowledge brokering such as informing policy deliberations, facilitating stakeholder communication, or identifying gaps in evidence. While less concrete in nature, these findings align with evidence that suggests that intangible effects of research on policy or practice are more common than direct effects [54], and highlight a key challenge in measuring the impact of KBs.

### Challenges in measuring the impact of a KB

Measuring the impact of KBs is a challenging process exacerbated by the fact that some KBs are ‘unwilling to claim personal responsibility for achievements’ (p. 8) resulting from their efforts [15]. Instead, some brokers suggested that their impact was focused on facilitating the process and building capacity and that the resulting outcomes (e.g. policy changes) were best attributed to the team with whom the broker interacted. In effect, KBs serve as the catalyst for change in how stakeholders acquire, interpret, and apply information. In order to effect this change, KBs must navigate contextually sensitive environments and negotiate timely and feasible responses to diverse stakeholder needs. In seeking to evaluate the impact of these varied KB activities, one must account for myriad contextual factors, which invariably complicate the measurement process.

Similarly, some have questioned the appropriateness of using empirical designs to evaluate the effectiveness of KT strategies (e.g. knowledge brokering) [46]. Of particular concern is the inability to account for all differences (e.g. personal, organizational) between participant sites. This measurement limitation arises from the real-world context in which KBs operate and poses interpretive challenges as it often remains unclear as to whether an observed outcome represents the true impact of the KB (i.e. treatment effect) or of some other factor. Further, differences in personal and organizational factors may moderate or conceal the effect of a KB intervention. Consequently, additional research is needed to better understand the individual attributes and contextual factors that may impact the effectiveness of KB strategies in health-related settings. In particular, methodologically rigourous case studies, qualitative designs (e.g. grounded theory), and mixed methods approaches may permit a more robust understanding of not only *if* KB strategies are effective, but also *under which circumstances* they will have the greatest likelihood of producing a significant and positive impact.

#### Limitations

Owing to the heterogeneous terminology and myriad role descriptions of KBs (e.g. ‘education facilitators’), discerning which studies to include proved challenging at times. However, all inclusion and exclusion decisions were reached through consensus among reviewers. Second, while we aimed to be inclusive in our characterization of KB activities and tasks, we did not contact study authors or the KBs who performed the reported interventions. Thus, it is possible that KBs may have performed activities not captured in this review. Given that this review did not aim to generate a comprehensive taxonomy of all possible brokering activities, we believe that the current description is appropriate. Third, measuring the effectiveness of KBs was marked by several challenges owing to the manner in which we defined evidence of ‘effectiveness’ (i.e. changes in knowledge, skills, policy/practice), the number of studies reporting outcome data, and the diverse real-world settings in which KBs operated. Ultimately, we found insufficient evidence to draw conclusions regarding the effectiveness of KBs in health-related settings.

## Conclusions

KBs represent the human component of KT strategies as they work collaboratively with stakeholders to facilitate the transfer and exchange of information in contextually diverse settings. In exploring how KBs operated in practice, we found that the activities and tasks which comprised these roles corresponded to the proposed characterization of KBs as knowledge managers, linkage agents, and capacity builders and that these roles often overlapped. Our findings also revealed significant heterogeneity in the settings, interventions, and role descriptions of the brokers. In assessing the effectiveness of KBs in practice, we explored reported changes in knowledge, skills, policies, and practices related to the KB interventions; however, owing to the limited availability of methodologically rigourous outcome data, findings were inconclusive. Accordingly, researchers are encouraged to report measurable outcomes of KB interventions in order to establish rigorous evidence of their effect before widespread use is encouraged.

## Additional files

Additional file 1:
**Literature search strategies and results.** (http://www.implementationscience.com/imedia/2014283521702996/supp1.pdf). (PDF 361 kb) Additional file 2:
**Grey literature search strategies and results.** (http://www.implementationscience.com/imedia/1230909141170299/supp2.pdf). (PDF 361 kb) Additional file 3:
**Summary of KB characteristics.** (http://www.implementationscience.com/imedia/5868567011931448/supp3.pdf). (PDF 237 kb) Additional file 4:
**Summary of MetaQAT appraisals.** (http://www.implementationscience.com/imedia/1334272237170299/supp4.pdf). (PDF 352 kb)

## Abbreviations

CFHI: Canadian Foundation for Healthcare Improvement
CHSRF: Canadian Health Services Research Foundation
KB: knowledge broker
KT: knowledge translation
MetaQAT: Meta Quality Appraisal Tool
RSS: really simple syndication
WHO: World Health Organization
