# Erratum to: ‘Exploring the function and effectiveness of knowledge brokers as facilitators of knowledge translation in health related settings: a systematic review and thematic analysis’

**DOI:** 10.1186/s13012-015-0358-2

**Published:** 2015-12-12

**Authors:** Catherine C. Bornbaum, Kathy Kornas, Leslea Peirson, Laura C. Rosella

**Affiliations:** Dalla Lana School of Public Health, University of Toronto, 155 College Street, 6th Floor, Toronto, M5T 3M7 ON Canada; Health & Rehabilitation Sciences, Western University, Elborn College, Room 2200, London, N6A 1H1 ON Canada; Public Health Ontario, Santé publique Ontario, 480 University Avenue, Suite 300, Toronto, M5G 1V2 ON Canada; McMaster Evidence Review and Synthesis Centre, School of Nursing, McMaster University Faculty of Health Sciences, 1280 Main St. W., Hamilton, L8S 4L8 ON Canada; Institute for Clinical Evaluative Sciences (ICES), G1 06, 2075 Bayview Avenue, Toronto, M4N 3M5 ON Canada

In viewing the paper [1] online, the author noticed a few errors/issues with the document. The image for Table 1 does not appear visible when selected. Table 1 also appears to have several horizontal lines missing from it under the subheader “Network development, maintenance, and facilitation”, and several vertical lines missing between the “knowledge management” and “linkage and exchange” activity domains which has been corrected here.

**Table 1:**
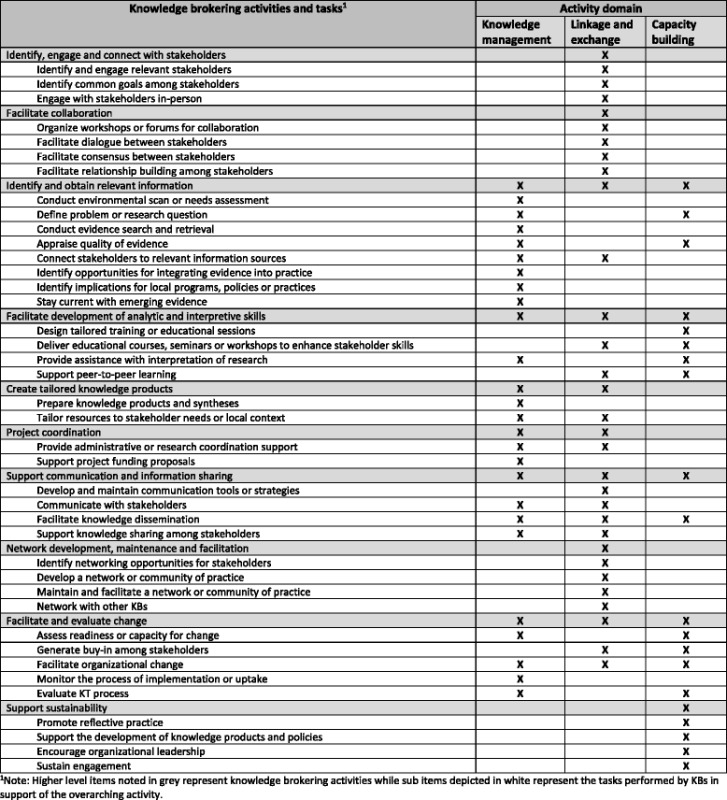
Classification of knowledge brokering tasks according to activity domains

Additionally, the wrong files have been uploaded for “Additional file 3” and “Additional file 4”. Currently the reference numbers in these additional files do not align with the final version of the manuscript. The correct files have been placed here.

## Additional files

Additional file 3:
**Summary of characteristics from studies investigating the use of knowledge brokers in** **health-related** **settings.** (PDF 238 kb) Additional file 4:
**Summary of MetaQAT appraisals from studies investigating the use of knowledge brokers in** **health-related** **settings.** (PDF 353 kb)
