# Supplementary material for: Exploring the function and effectiveness of knowledge brokers as facilitators of knowledge translation in health-related settings: a systematic review and thematic analysis
Source: Implement Sci. 2015 Nov 20;10:162. doi: 10.1186/s13012-015-0351-9 (PMC4653833; doi:10.1186/s13012-015-0351-9)
Supplement: Additional file 1: — Literature search strategies and results. (http://www.implementationscience.com/imedia/2014283521702996/supp1.pdf). (PDF 361 kb) [file 13012_2015_351_MOESM1_ESM.pdf]

Knowledge brokering review

Databases:

- MEDLINE (Ovid platform)
- Embase (Ovid platform)
- PsycINFO (Ovid platform)
- CINAHL (EBSCOhost platform)
- ERIC (ProQuest platform)
- Scopus (ScienceDirect platform)
- SocINDEX (EBSCOhost platform)
- Health Business Elite (EBSCOhost platform)

Search strategies:

NB: Preliminary search conducted January 3, 2014.

MEDLINE 1946 to Present; MEDLINE In-Process & Other Non-Indexed Citations

#	Searches	Results
1	Knowledge Management/ or Information Dissemination/ or Translational Medical Research/ or Interdisciplinary Communication/ or Technology Transfer/	26769
2	Professional Role/ or Capacity Building/ or Administrative Personnel/ or Leadership/	45072
3	1 and 2	824
4	("know-do" or ((knowledge$ or research$ or information$ or evidence$ or science or findings) adj2 (translat$ or transfer$ or exchang$ or action$ or practice$ or decision$ or implement$ or manag$ or disseminat$ or appl$ or share$ or sharing or uptak$ or "use" or utilis$ or utiliz$ or mobilis$ or mobiliz$ or integrat$ or communicat$ or adopt$)) or "technology transfer").ti,ab,kf.	150018
5	(((knowledge$ or research$ or information$ or evidence$ or science or findings) adj3 (broker$ or go-between$ or intermediar$ or liaison$ or mediator$ or navigator$ or officer$ or translator$ or facilitator$ or leader or leaders)) or (change adj2 agent$) or (boundary adj1 spanner) or (opinion adj1 leader$) or (linkage adj1 agent$) or "researcher-practitioner" or "researcher-practitioners" or (structural adj1 broker$) or (capacity adj1 builder$)).ti,ab,kf.	4901
6	4 and 5	954
7	((knowledge adj3 broker$) or (knowledge adj1 manager$)).ti,ab,kf.	149
8	3 or 6 or 7	1816
9	limit 8 to english language	1718
10	remove duplicates from 9	1530

Embase 1974 to 2013 Week 52

#	Searches	Results
1	knowledge management/ or information dissemination/ or translational research/ or interdisciplinary communication/ or professional knowledge/	34848
2	professional standard/ or capacity building/ or administrative personnel/ or leadership/	75995

**3**	1 and 2	1787
**4**	("know-do" or ((knowledge$ or research$ or information$ or evidence$ or science or findings) adj2 (translat$ or transfer$ or exchang$ or action$ or practice$ or decision$ or implement$ or manag$ or disseminat$ or appl$ or share$ or sharing or uptak$ or "use" or utilis$ or utiliz$ or mobilis$ or mobiliz$ or integrat$ or communicat$ or adopt$)) or "technology transfer").ti,ab,kw.	175656
**5**	(((knowledge$ or research$ or information$ or evidence$ or science or findings) adj3 (broker$ or go-between$ or intermediar$ or liaison$ or mediator$ or navigator$ or officer$ or translator$ or facilitator$ or leader or leaders)) or (change adj2 agent$) or (boundary adj1 spanner) or (opinion adj1 leader$) or (linkage adj1 agent$) or "researcher-practitioner" or "researcher-practitioners" or (structural adj1 broker$) or (capacity adj1 builder$)).ti,ab,kw.	5600
**6**	4 and 5	1018
**7**	((knowledge adj3 broker$) or (knowledge adj1 manager$)).ti,ab,kw.	164
**8**	3 or 6 or 7	2868
**9**	limit 8 to english language	2739
**10**	limit 9 to exclude medline journals	298

PsycINFO 1967 to December Week 4 2013 (run simultaneously in Ovid MEDLINE(R) In-Process & Other Non-Indexed Citations and Ovid MEDLINE(R) 1946 to Present to remove duplicate records)

#	Searches	Results
**1**	exp Knowledge Management/ or exp Knowledge Transfer/ or exp Knowledge Representation/ or exp Information Dissemination/ or Technology Transfer/	73039
**2**	exp Management Personnel/ or Professional Personnel/ or Professional Identity/ or Roles/ or exp Leadership/	222711
**3**	1 and 2	899
**4**	("know-do" or ((knowledge$ or research$ or information$ or evidence$ or science or findings) adj2 (translat$ or transfer$ or exchang$ or action$ or practice$ or decision$ or implement$ or manag$ or disseminat$ or appl$ or share$ or sharing or uptak$ or "use" or utilis$ or utiliz$ or mobilis$ or mobiliz$ or integrat$ or communicat$ or adopt$)) or "technology transfer").ti,ab,id.	266400
**5**	(((knowledge$ or research$ or information$ or evidence$ or science or findings) adj3 (broker$ or go-between$ or intermediar$ or liaison$ or mediator$ or navigator$ or officer$ or translator$ or facilitator$ or leader or leaders)) or (change adj2 agent$) or (boundary adj1 spanner) or (opinion adj1 leader$) or (linkage adj1 agent$) or "researcher-practitioner" or "researcher-practitioners" or (structural adj1 broker$) or (capacity adj1 builder$)).ti,ab,id.	10661
**6**	4 and 5	1951
**7**	((knowledge adj3 broker$) or (knowledge adj1 manager$)).ti,ab,id.	338
**8**	3 or 6 or 7	3006
**9**	limit 8 to english language	2934
**10**	remove duplicates from 9	2608
**11**	10 use psyb	1243

CINAHL with Full Text

#	Query	Results
**S1**	(MH "Knowledge Management") OR (MH "Selective Dissemination of Information")	1,099
**S2**	(MH "Professional Role") OR (MH "Administrative Personnel") OR (MH "Management") OR (MH "Leadership")	47,355
**S3**	S1 AND S2	114
**S4**	TI ("know-do" or ((knowledge* or research* or information* or evidence* or science or findings) N2 (translat* or transfer* or exchang* or action* or practice* or decision* or implement* or manag* or disseminat* or appl* or share* or sharing or uptak* or "use" or utilis* or utiliz* or mobilis* or mobiliz* or integrat* or communicat* or adopt*)) or "technology transfer") OR AB ("know-do" or ((knowledge* or research* or information* or evidence* or science or findings) N2 (translat* or transfer* or exchang* or action* or practice* or decision* or implement* or manag* or disseminat* or appl* or share* or sharing or uptak* or "use" or utilis* or utiliz* or mobilis* or mobiliz* or integrat* or communicat* or adopt*)) or "technology transfer")	75,539
**S5**	TI (((knowledge* or research* or information* or evidence* or science or findings) N2 (broker* or go-between* or intermediar* or liaison* or mediator* or navigator* or officer* or translator* or facilitator* or leader or leaders)) or (change N1 agent*) or (boundary N0 spanner) or (opinion N0 leader*) or (linkage N0 agent*) or "researcher-practitioner" or "researcher-practitioners" or (structural N0 broker*) or (capacity N0 builder*)) OR AB (((knowledge* or research* or information* or evidence* or science or findings) N2 (broker* or go-between* or intermediar* or liaison* or mediator* or navigator* or officer* or translator* or facilitator* or leader or leaders)) or (change N1 agent*) or (boundary N0 spanner) or (opinion N0 leader*) or (linkage N0 agent*) or "researcher-practitioner" or "researcher-practitioners" or (structural N0 broker*) or (capacity N0 builder*))	1,909
**S6**	S4 AND S5	535
**S7**	TI ((knowledge N3 broker*) OR (knowledge N1 manager*)) OR AB ((knowledge N3 broker*) OR (knowledge N1 manager*))	100
**S8**	S3 OR S6 OR S7<br>Limiters - English Language; Exclude MEDLINE records	290

ERIC

#	Searches	Results
**1**	(SU.EXACT("Selective Dissemination of Information") OR SU.EXACT("Research Utilization") OR SU.EXACT.EXPLODE("Information Management") OR SU.EXACT("Information Utilization") OR SU.EXACT("Information Dissemination") OR SU.EXACT("Evaluation Utilization") OR SU.EXACT.EXPLODE("Knowledge Management") OR SU.EXACT.EXPLODE("Adoption (Ideas)") OR SU.EXACT.EXPLODE("Information Transfer") OR SU.EXACT.EXPLODE("Technology Transfer")) OR (SU.EXACT("Decision Making") AND SU.EXACT("Evidence"))	26,016
**2**	SU.EXACT("Change Agents") OR SU.EXACT("Leaders") OR SU.EXACT("Capacity Building") OR SU.EXACT("Leadership") OR SU.EXACT("Role") OR SU.EXACT("Managerial Occupations") OR SU.EXACT("Professional Occupations") OR SU.EXACT("Staff Role") OR SU.EXACT("Administrator Role") OR SU.EXACT("Administrators") OR SU.EXACT("Leadership Role")	66,238
**3**	1 and 2	1,741

**4**	"know-do" or ((knowledge* or research* or information* or evidence* or science or findings) N/2 (translat* or transfer* or exchang* or action* or practice* or decision* or implement* or manag* or disseminat* or appl* or share* or sharing or uptak* or "use" or utilis* or utiliz* or mobilis* or mobiliz* or integrat* or communicat* or adopt*)) or "technology transfer"	141,382
**5**	((knowledge* or research* or information* or evidence* or science or findings) N/2 (broker* or go-between* or intermediar* or liaison* or mediator* or navigator* or officer* or translator* or facilitator* or leader or leaders)) or (change N/1 agent*) or (boundary N/0 spanner) or (opinion N/0 leader*) or (linkage N/0 agent*) or "researcher-practitioner" or "researcher-practitioners" or (structural N/0 broker*) or (capacity N/0 builder*)	9,906
**6**	4 and 5	2,018
**7**	health* or medic* or hospital* or care or therap* or clinic* or doctor* or physician* or nurs* or epidemiolog* or disease* or illness* or infect*	242,513
**8**	(3 or 6) and 7	423
**9**	(knowledge N/2 broker*) OR (knowledge N/0 manager*)	76
**10**	8 or 9	486

Scopus

#	Query	Results
**1**	TITLE({know-do} OR ((knowledge* OR research* OR information* OR evidence* OR science OR findings) W/2 (translat* OR transfer* OR exchang* OR action* OR practice* OR decision* OR implement* OR manag* OR disseminat* OR appl* OR share* OR sharing OR uptak* OR "use" OR utilis* OR utiliz* OR mobilis* OR mobiliz* OR integrat* OR communicat* OR adopt*)) OR (technology W/0 transfer)) OR KEY({know-do} OR ((knowledge* OR research* OR information* OR evidence* OR science OR findings) W/2 (translat* OR transfer* OR exchang* OR action* OR practice* OR decision* OR implement* OR manag* OR disseminat* OR appl* OR share* OR sharing OR uptak* OR "use" OR utilis* OR utiliz* OR mobilis* OR mobiliz* OR integrat* OR communicat* OR adopt*)) OR (technology W/0 transfer))	720,235
**2**	TITLE-ABS-KEY(((knowledge* OR research* OR information* OR evidence* OR science OR findings) W/3 (broker* OR go-between* OR intermediar* OR liaison* OR mediator* OR navigator* OR officer* OR translator* OR facilitator* OR leader OR leaders)) OR (change W/1 agent*) OR (boundary W/0 spanner) OR (opinion W/0 leader*) OR (linkage W/0 agent*) OR {researcher-practitioner} OR {researcher-practitioners} OR (structural W/0 broker*) OR (capacity W/0 builder*))	19,038
**3**	#1 AND #2	3,604
**4**	TITLE-ABS-KEY((knowledge W/2 broker*) OR (knowledge W/0 manager*))	892
**5**	#3 OR #4	4,272
**6**	TITLE-ABS-KEY(health* OR medic* OR hospital* OR care OR therap* OR clinic* OR doctor* OR physician* OR nurs* OR epidemiolog* OR disease* OR illness* OR infect*)	15,834,576
**7**	#5 AND #6	1,595
**8**	7 AND (LIMIT-TO(LANGUAGE, "English"))	1,554

SocINDEX with Full Text

#	Query	Results
**S1**	"know-do" or ((knowledge* or research* or information* or evidence* or science or	101,457

	findings) N2 (translat* or transfer* or exchang* or action* or practice* or decision* or implement* or manag* or disseminat* or appl* or share* or sharing or uptak* or "use" or utilis* or utiliz* or mobilis* or mobiliz* or integrat* or communicat* or adopt*)) or "technology transfer"	
**S2**	((knowledge* or research* or information* or evidence* or science or findings) N2 (broker* or go-between* or intermediar* or liaison* or mediator* or navigator* or officer* or translator* or facilitator* or leader or leaders)) or (change N1 agent*) or (boundary N0 spanner) or (opinion N0 leader*) or (linkage N0 agent*) or "researcher-practitioner" or "researcher-practitioners" or (structural N0 broker*) or (capacity N0 builder*)	4,763
**S3**	S1 AND S2	756
**S4**	(knowledge N3 broker*) OR (knowledge N0 manager*)	95
**S5**	S3 OR S4	816
**S6**	S3 OR S4<br>Limiters - Language: Afrikaans, Catalan, Chinese, Croatian, Czech, Danish, Dutch/Flemish, Finnish, French, German, Galician, Hungarian, Italian, Japanese, Lithuanian, Norwegian, Polish, Portuguese, Romanian, Russian, Slovak, Slovenian, Spanish, Swedish, Turkish	14
**S7**	S5 NOT S6	802

Health Business Elite

#	Query	Results
**S1**	"know-do" or ((knowledge* or research* or information* or evidence* or science or findings) N2 (translat* or transfer* or exchang* or action* or practice* or decision* or implement* or manag* or disseminat* or appl* or share* or sharing or uptak* or "use" or utilis* or utiliz* or mobilis* or mobiliz* or integrat* or communicat* or adopt*)) or "technology transfer"	105,407
**S2**	((knowledge* or research* or information* or evidence* or science or findings) N2 (broker* or go-between* or intermediar* or liaison* or mediator* or navigator* or officer* or translator* or facilitator* or leader or leaders)) or (change N1 agent*) or (boundary N0 spanner) or (opinion N0 leader*) or (linkage N0 agent*) or "researcher-practitioner" or "researcher-practitioners" or (structural N0 broker*) or (capacity N0 builder*)	17,722
**S3**	S1 AND S2	3,002
**S4**	health* OR medic* OR hospital* OR care OR therap* OR clinic* OR doctor* OR physician* OR nurs* OR epidemiolog* OR disease* OR illness* OR infect*	754,361
**S5**	S3 AND S4	704
**S6**	(knowledge N3 broker*) OR (knowledge N0 manager*)	118
**S7**	S5 OR S6	819

Search yield:

- Gross yield: 7022
  - MEDLINE: 1530
  - Embase: 298
  - PsycINFO: 1243
  - CINAHL: 290
  - ERIC: 486

    - Scopus: 1554
    - SocINDEX: 802
    - Health Business Elite: 819
- Duplicates: 999

UPDATED SEARCH (JANUARY – NOVEMBER 2014)

Databases:

- MEDLINE (Ovid platform)
- Embase (Ovid platform)
- PsycINFO (Ovid platform)
- CINAHL (EBSCOhost platform)
- ERIC (Ovid platform)
- Scopus (ScienceDirect platform)
- SocINDEX (EBSCOhost platform)
- Health Business Elite (EBSCOhost platform)

Search strategies:

NB: Follow-up search conducted November 24, 2014.

MEDLINE 1946 to Present; MEDLINE In-Process & Other Non-Indexed Citations

#	Searches	Results
1	Knowledge Management/ or Information Dissemination/ or Translational Medical Research/ or Interdisciplinary Communication/ or Technology Transfer/	29113
2	Professional Role/ or Capacity Building/ or Administrative Personnel/ or Leadership/	47319
3	1 and 2	889
4	("know-do" or ((knowledge$ or research$ or information$ or evidence$ or science or findings) adj2 (translat$ or transfer$ or exchang$ or action$ or practice$ or decision$ or implement$ or manag$ or disseminat$ or appl$ or share$ or sharing or uptak$ or "use" or utilis$ or utiliz$ or mobilis$ or mobiliz$ or integrat$ or communicat$ or adopt$)) or "technology transfer").ti,ab,kf.	156501
5	(((knowledge$ or research$ or information$ or evidence$ or science or findings) adj3 (broker$ or go-between$ or intermediar$ or liaison$ or mediator$ or navigator$ or officer$ or translator$ or facilitator$ or leader or leaders)) or (change adj2 agent$) or (boundary adj1 spanner) or (opinion adj1 leader$) or (linkage adj1 agent$) or "researcher-practitioner" or "researcher-practitioners" or (structural adj1 broker$) or (capacity adj1 builder$)).ti,ab,kf.	5035
6	4 and 5	970
7	((knowledge adj3 broker$) or (knowledge adj1 manager$)).ti,ab,kf.	159
8	3 or 6 or 7	1914
9	limit 8 to english language	1799
10	remove duplicates from 9	1723
11	limit 10 to yr="2014 -Current"	132

Embase 1974 to 2014 November 20

#	Searches	Results
1	knowledge management/ or information dissemination/ or translational research/ or interdisciplinary communication/ or professional knowledge/	39002
2	professional standard/ or capacity building/ or administrative personnel/ or leadership/	81355
3	1 and 2	1983
4	("know-do" or ((knowledge$ or research$ or information$ or evidence$ or science or findings) adj2 (translat$ or transfer$ or exchang$ or action$ or practice$ or decision$ or implement$ or manag$ or disseminat$ or appl$ or share$ or sharing or uptak$ or "use" or utilis$ or utiliz$ or mobilis$ or mobiliz$ or integrat$ or communicat$ or adopt$)) or "technology transfer").ti,ab,kw.	188297
5	(((knowledge$ or research$ or information$ or evidence$ or science or findings) adj3 (broker$ or go-between$ or intermediar$ or liaison$ or mediator$ or navigator$ or officer$ or translator$ or facilitator$ or leader or leaders)) or (change adj2 agent$) or (boundary adj1 spanner) or (opinion adj1 leader$) or (linkage adj1 agent$) or "researcher-practitioner" or "researcher-practitioners" or (structural adj1 broker$) or (capacity adj1 builder$)).ti,ab,kw.	5931
6	4 and 5	1121
7	((knowledge adj3 broker$) or (knowledge adj1 manager$)).ti,ab,kw.	192
8	3 or 6 or 7	3172
9	limit 8 to english language	3027
10	limit 9 to exclude medline journals	334
11	limit 10 to yr="2014 -Current"	30

PsycINFO 1967 to November Week 3 2014 (run simultaneously in Ovid MEDLINE(R) In-Process & Other Non-Indexed Citations and Ovid MEDLINE(R) 1946 to Present to remove duplicate records)

Ovid MEDLINE(R), PsycINFO

#	Searches	Results
1	exp Knowledge Management/ or exp Knowledge Transfer/ or exp Knowledge Representation/ or exp Information Dissemination/ or Technology Transfer/	78178
2	exp Management Personnel/ or Professional Personnel/ or Professional Identity/ or Roles/ or exp Leadership/	232603
3	1 and 2	962
4	("know-do" or ((knowledge$ or research$ or information$ or evidence$ or science or findings) adj2 (translat$ or transfer$ or exchang$ or action$ or practice$ or decision$ or implement$ or manag$ or disseminat$ or appl$ or share$ or sharing or uptak$ or "use" or utilis$ or utiliz$ or mobilis$ or mobiliz$ or integrat$ or communicat$ or adopt$)) or "technology transfer").ti,ab,id.	283999
5	(((knowledge$ or research$ or information$ or evidence$ or science or findings) adj3 (broker$ or go-between$ or intermediar$ or liaison$ or mediator$ or navigator$ or officer$ or translator$ or facilitator$ or leader or leaders)) or (change adj2 agent$) or (boundary adj1 spanner) or (opinion adj1 leader$) or (linkage adj1 agent$) or "researcher-practitioner" or "researcher-practitioners" or (structural adj1 broker$) or (capacity adj1 builder$)).ti,ab,id.	11348
6	4 and 5	2108
7	((knowledge adj3 broker$) or (knowledge adj1 manager$)).ti,ab,id.	377

**8**	3 or 6 or 7	3250
**9**	limit 8 to english language	3172
**10**	remove duplicates from 9	2902
**11**	10 use psyb	1411
**12**	limit 11 to yr="2014 -Current"	124

CINAHL with Full Text

#	Query	Limiters/Expanders	Results
**S8**	S3 OR S6 OR S7	Limiters - Published Date: 20140101-20141231; English Language; Exclude MEDLINE records Search modes - Boolean/Phrase	15
**S7**	TI ((knowledge N3 broker*) OR (knowledge N1 manager*)) OR AB ((knowledge N3 broker*) OR (knowledge N1 manager*))	Search modes - Boolean/Phrase	111
**S6**	S4 AND S5	Search modes - Boolean/Phrase	571
**S5**	TI (((knowledge* or research* or information* or evidence* or science or findings) N2 (broker* or go-between* or intermediar* or liaison* or mediator* or navigator* or officer* or translator* or facilitator* or leader or leaders)) or (change N1 agent*) or (boundary N0 spanner) or (opinion N0 leader*) or (linkage N0 agent*) or "researcher-practitioner" or "researcher-practitioners" or (structural N0 broker*) or (capacity N0 builder*)) OR AB (((knowledge* or research* or information* or evidence* or science or findings) N2 (broker* or go-between* or intermediar* or liaison* or mediator* or navigator* or officer* or translator* or facilitator* or leader or leaders)) or (change N1 agent*) or (boundary N0 spanner) or (opinion N0 leader*) or (linkage N0 agent*) or "researcher-practitioner" or "researcher-practitioners" or (structural N0 broker*) or (capacity N0 builder*))	Search modes - Boolean/Phrase	2,007
**S4**	TI ("know-do" or ((knowledge* or research* or information* or evidence* or science or findings) N2 (translat* or transfer* or exchang* or action* or practice* or decision* or implement* or manag* or disseminat* or appl* or share* or sharing or uptak* or "use" or utilis* or utiliz* or mobilis* or mobiliz* or integrat* or communicat* or adopt*)) or "technology transfer") OR AB ("know-do" or ((knowledge* or	Search modes - Boolean/Phrase	80,715

	research* or information* or evidence* or science or findings) N2 (translat* or transfer* or exchang* or action* or practice* or decision* or implement* or manag* or disseminat* or appl* or share* or sharing or uptak* or "use" or utilis* or utiliz* or mobilis* or mobiliz* or integrat* or communicat* or adopt*)) or "technology transfer")		
**S3**	S1 AND S2	Search modes - Boolean/Phrase	128
**S2**	(MH "Professional Role") OR (MH "Administrative Personnel") OR (MH "Management") OR (MH "Leadership")	Search modes - Boolean/Phrase	51,004
**S1**	(MH "Knowledge Management") OR (MH "Selective Dissemination of Information")	Search modes - Boolean/Phrase	1,197

ERIC

#	Searches	Results
**1**	("Selective Dissemination of Information"/ or Research Utilization/ or exp Information Management/ or Information Utilization/ or Information Dissemination/ or Evaluation Utilization/ or exp Knowledge Management/ or exp "Adoption (Ideas)"/ or exp Information Transfer/ or exp Technology Transfer/ or Decision Making/) and Evidence/	364
**2**	Change Agents/ or Leaders/ or Capacity Building/ or Leadership/ or Role/ or Managerial Occupations/ or Professional Occupations/ or Staff Role/ or Administrator Role/ or Administrators/ or Leadership Role/	50717
**3**	1 and 2	35
**4**	("know-do" or ((knowledge* or research* or information* or evidence* or science or findings) adj3 (translat* or transfer* or exchang* or action* or practice* or decision* or implement* or manag* or disseminat* or appl* or share* or sharing or uptak* or "use" or utilis* or utiliz* or mobilis* or mobiliz* or integrat* or communicat* or adopt*)) or "technology transfer").mp.	115723
**5**	(((knowledge* or research* or information* or evidence* or science or findings) adj3 (broker* or go-between* or intermediar* or liaison* or mediator* or navigator* or officer* or translator* or facilitator* or leader or leaders)) or (change adj2 agent*) or (boundary adj1 spanner) or (opinion adj1 leader*) or (linkage adj1 agent*) or "researcher-practitioner" or "researcher-practitioners" or (structural adj1 broker*) or (capacity adj1 builder*)).mp.	9558
**6**	4 and 5	1729
**7**	(health* or medic* or hospital* or care or therap* or clinic* or doctor* or physician* or nurs* or epidemiolog* or disease* or illness* or infect*).mp. [mp=abstract, title, heading word, identifiers]	202213
**8**	3 or 6	1756
**9**	7 and 8	188
**10**	((knowledge adj3 broker*) or (knowledge adj1 manager*)).mp.	74
**11**	9 or 10	256
**12**	limit 11 to yr="2014 -Current"	4

Scopus

#	Query	Results
**1**	TITLE({know-do} OR ((knowledge* OR research* OR information* OR evidence* OR science OR findings) W/2 (translat* OR transfer* OR exchang* OR action* OR practice* OR decision* OR implement* OR manag* OR disseminat* OR appl* OR share* OR sharing OR uptak* OR "use" OR utilis* OR utiliz* OR mobilis* OR mobiliz* OR integrat* OR communicat* OR adopt*)) OR (technology W/0 transfer)) OR KEY({know-do} OR ((knowledge* OR research* OR information* OR evidence* OR science OR findings) W/2 (translat* OR transfer* OR exchang* OR action* OR practice* OR decision* OR implement* OR manag* OR disseminat* OR appl* OR share* OR sharing OR uptak* OR "use" OR utilis* OR utiliz* OR mobilis* OR mobiliz* OR integrat* OR communicat* OR adopt*)) OR (technology W/0 transfer))	785,392
**2**	TITLE-ABS-KEY(((knowledge* OR research* OR information* OR evidence* OR science OR findings) W/3 (broker* OR go-between* OR intermediar* OR liaison* OR mediator* OR navigator* OR officer* OR translator* OR facilitator* OR leader OR leaders)) OR (change W/1 agent*) OR (boundary W/0 spanner) OR (opinion W/0 leader*) OR (linkage W/0 agent*) OR {researcher-practitioner} OR {researcher-practitioners} OR (structural W/0 broker*) OR (capacity W/0 builder*))	21,329
**3**	#1 AND #2	4,080
**4**	TITLE-ABS-KEY((knowledge W/2 broker*) OR (knowledge W/0 manager*))	1,045
**5**	#3 OR #4	4,855
**6**	TITLE-ABS-KEY(health* OR medic* OR hospital* OR care OR therap* OR clinic* OR doctor* OR physician* OR nurs* OR epidemiolog* OR disease* OR illness* OR infect*)	16,611,101
**7**	#5 AND #6	1,805
**8**	7 AND (LIMIT-TO (PUBYEAR , 2015) OR LIMIT-TO (PUBYEAR , 2014)) AND (LIMIT-TO (LANGUAGE , "English")	129

SocINDEX with Full Text

#	Query	Limiters/Expanders	Results
**S8**	S5 NOT S6	Limiters - Date of Publication: 20140101-20141231<br>Search modes - Boolean/Phrase	22
**S7**	S5 NOT S6	Search modes - Boolean/Phrase	831
**S6**	S3 OR S4	Limiters - Language: Afrikaans, Catalan, Chinese, Croatian, Czech, Danish, Dutch/Flemish, Finnish, French, German, Galician, Hungarian, Italian, Japanese, Lithuanian, Norwegian, Polish, Portuguese, Romanian, Russian, Slovak, Slovenian, Spanish, Swedish, Turkish<br>Search modes -	13

		Boolean/Phrase	
**S5**	S3 OR S4	Search modes - Boolean/Phrase	844
**S4**	(knowledge N3 broker*) OR (knowledge N0 manager*)	Search modes - Boolean/Phrase	104
**S3**	S1 AND S2	Search modes - Boolean/Phrase	781
**S2**	((knowledge* or research* or information* or evidence* or science or findings) N2 (broker* or go-between* or intermediar* or liaison* or mediator* or navigator* or officer* or translator* or facilitator* or leader or leaders)) or (change N1 agent*) or (boundary N0 spanner) or (opinion N0 leader*) or (linkage N0 agent*) or "researcher-practitioner" or "researcher-practitioners" or (structural N0 broker*) or (capacity N0 builder*)	Search modes - Boolean/Phrase	4,888
**S1**	"know-do" or ((knowledge* or research* or information* or evidence* or science or findings) N2 (translat* or transfer* or exchang* or action* or practice* or decision* or implement* or manag* or disseminat* or appl* or share* or sharing or uptak* or "use" or utilis* or utiliz* or mobilis* or mobiliz* or integrat* or communicat* or adopt*)) or "technology transfer"	Search modes - Boolean/Phrase	104,663

Health Business Elite

	Query	Limiters/Expanders	Results
**S8**	S5 OR S6	Limiters - Published Date: 20140101-20141231<br>Search modes - Boolean/Phrase	46
**S7**	S5 OR S6	Search modes - Boolean/Phrase	887
**S6**	(knowledge N3 broker*) OR (knowledge N0 manager*)	Search modes - Boolean/Phrase	121
**S5**	S3 AND S4	Search modes - Boolean/Phrase	769
**S4**	health* OR medic* OR hospital* OR care OR therap* OR clinic* OR doctor* OR physician* OR nurs* OR epidemiolog* OR disease* OR illness* OR infect*	Search modes - Boolean/Phrase	783,564
**S3**	S1 AND S2	Search modes - Boolean/Phrase	3,109
**S2**	((knowledge* or research* or information* or evidence* or science or findings) N2 (broker* or go-between* or intermediar* or liaison* or mediator* or navigator* or officer* or translator* or facilitator* or	Search modes - Boolean/Phrase	17,949

	leader or leaders)) or (change N1 agent*) or (boundary N0 spanner) or (opinion N0 leader*) or (linkage N0 agent*) or "researcher-practitioner" or "researcher-practitioners" or (structural N0 broker*) or (capacity N0 builder*)		
**S1**	"know-do" or ((knowledge* or research* or information* or evidence* or science or findings) N2 (translat* or transfer* or exchang* or action* or practice* or decision* or implement* or manag* or disseminat* or appl* or share* or sharing or uptak* or "use" or utilis* or utiliz* or mobilis* or mobiliz* or integrat* or communicat* or adopt*)) or "technology transfer"	Search modes - Boolean/Phrase	108,127

Search yield (January – November 2014):

- Gross yield: 502
  - MEDLINE: 132
  - Embase: 30
  - PsycINFO: 124
  - CINAHL: 15
  - ERIC: 4
  - Scopus: 129
  - SocINDEX: 22
  - Health Business Elite: 46
- Duplicates: 70
- Net yield: 432
