# Supplementary material for: Exploring the function and effectiveness of knowledge brokers as facilitators of knowledge translation in health-related settings: a systematic review and thematic analysis
Source: Implement Sci. 2015 Nov 20;10:162. doi: 10.1186/s13012-015-0351-9 (PMC4653833; doi:10.1186/s13012-015-0351-9)
Supplement: Additional file 2: — Grey literature search strategies and results. (http://www.implementationscience.com/imedia/1230909141170299/supp2.pdf). (PDF 361 kb) [file 13012_2015_351_MOESM2_ESM.pdf]

Knowledge brokering review

Databases:

- Grey literature

Search strings:

("knowledge broker" OR "knowledge brokering" OR "knowledge brokerage") AND (health OR medical OR medicine OR nursing OR care)

"knowledge manager" AND (health OR medical OR medicine OR nursing OR care)

"linkage agent" AND (health OR medical OR medicine OR nursing OR care) AND (knowledge-translation OR knowledge-transfer OR knowledge-management OR knowledge-exchange OR knowledge-practice OR knowledge-action OR knowledge-utilization OR knowledge-sharing OR knowledge-mobilization

"capacity builder" AND (health OR medical OR medicine OR nursing OR care) AND (knowledge-translation OR knowledge-transfer OR knowledge-management OR knowledge-exchange OR knowledge-practice OR knowledge-action OR knowledge-utilization OR knowledge-sharing OR knowledge-mobilization)

Custom search engines:

Custom Search Engine for Ontario Public Health Unit Websites	http://www.ophla.ca/customsearch.htm
Custom Search Engine for Canadian Public Health Information	http://www.ophla.ca/customsearchcanada.htm

Core sources:

National Collaborating Centre for Methods and Tools	http://www.nccmt.ca/
Canadian Foundation for Healthcare Improvement (formerly the Canadian Health Services Research Foundation)	http://www.cfhi-fcass.ca/
Health Evidence	http://www.healthevidence.org/
Canadian Institutes of Health Research	http://www.cihr-irsc.gc.ca/
World Health Organization	http://www.who.int/
WHO Regional Office for Europe<br>See: BRIDGE (Brokering knowledge and Research Information to support the Development and Governance of health systems in Europe) policy brief and summary series	http://www.euro.who.int/

Knowledge Brokering Review – Grey Literature Search Results

SOURCE	SEARCH STRING	SOURCES RETRIEVED	APPLICABLE LINKS
**GREY LITERATURE**			
Custom Search Engine for Ontario Public Health Unit Websites http://www.ophla.ca/customsearch.htm	("knowledge broker" OR "knowledge brokering" OR "knowledge brokerage") AND (health OR medical OR medicine OR nursing OR care)	21	3 articles applicable for screening<br><br>http://www.ottawa.ca/calendar/ottawa/citycouncil/cpsc/2010/02-04/04%20-%20CURE_NPI_Final_Report_20081.pdf<br><br>http://archives.york.ca/councilcommitteearchives/pdf/rpt%2010%20cls%208-7.pdf<br><br>http://www.chnc.ca/documents/2009_conference/2009_-_A2_-_Fernane.pdf
	"knowledge manager" AND (health OR medical OR medicine OR nursing OR care)	1	0 applicable for screening
	"linkage agent" AND (health OR medical OR medicine OR nursing OR care) AND (knowledge-translation OR knowledge-transfer OR knowledge-management OR knowledge-exchange OR knowledge-practice OR knowledge-action OR knowledge-utilization OR knowledge-sharing OR knowledge-mobilization	0	0 applicable for screening
	"capacity builder" AND (health OR medical OR medicine OR nursing OR care) AND (knowledge-translation OR knowledge-transfer OR knowledge-management OR knowledge-exchange OR knowledge-practice OR knowledge-action OR knowledge-utilization OR knowledge-sharing OR knowledge-mobilization)	0	0 applicable for screening
Custom Search Engine for Canadian Public Health Information<br><br>http://www.o	("knowledge broker" OR "knowledge brokering" OR "knowledge brokerage") AND (health OR medical OR medicine OR nursing OR care)		Eliminated due to poor search stratification

SOURCE	SEARCH STRING	SOURCES RETRIEVED	APPLICABLE LINKS
phla.ca/customsearchcanada.htm			
	"knowledge manager" AND (health OR medical OR medicine OR nursing OR care)		Eliminated due to poor search stratification
	"linkage agent" AND (health OR medical OR medicine OR nursing OR care) AND (knowledge-translation OR knowledge-transfer OR knowledge-management OR knowledge-exchange OR knowledge-practice OR knowledge-action OR knowledge-utilization OR knowledge-sharing OR knowledge-mobilization		Eliminated due to poor search stratification
	"capacity builder" AND (health OR medical OR medicine OR nursing OR care) AND (knowledge-translation OR knowledge-transfer OR knowledge-management OR knowledge-exchange OR knowledge-practice OR knowledge-action OR knowledge-utilization OR knowledge-sharing OR knowledge-mobilization)		Eliminated due to poor search stratification
National Collaborating Centre for Methods and Tools<br><br>http://www.nccmt.ca/	("knowledge broker" OR "knowledge brokering" OR "knowledge brokerage") AND (health OR medical OR medicine OR nursing OR care)<br><br>Search string 1	60	1. http://www.nccmt.ca/registry/view/eng/160.html<br>2. http://www.nccmt.ca/registry/view/eng/167.html<br>3. http://www.nccmt.ca/registry/view/eng/139.html<br>4. http://www.nccmt.ca/registry/view/eng/170.html<br>5. http://www.nccmt.ca/pubs/transcripts/eiph07_transcript.pdf<br>6. http://www.nccmt.ca/registry/view/eng/204.html<br>7. http://www.nccmt.ca/registry/view/eng/110.html

SOURCE	SEARCH STRING	SOURCES RETRIEVED	APPLICABLE LINKS
			8. http://www.nccmt.ca/pubs/NCCMT_fall2008.pdf<br>9. http://www.nccmt.ca/pubs/KMpaper_EN.pdf<br>10. http://www.nccmt.ca/pubs/KMforumReport_EN.pdf
	"knowledge manager" AND (health OR medical OR medicine OR nursing OR care)	2	none
	"linkage agent" AND (health OR medical OR medicine OR nursing OR care) AND (knowledge-translation OR knowledge-transfer OR knowledge-management OR knowledge-exchange OR knowledge-practice OR knowledge-action OR knowledge-utilization OR knowledge-sharing OR knowledge-mobilization	0	
	"capacity builder" AND (health OR medical OR medicine OR nursing OR care) AND (knowledge-translation OR knowledge-transfer OR knowledge-management OR knowledge-exchange OR knowledge-practice OR knowledge-action OR knowledge-utilization OR knowledge-sharing OR knowledge-mobilization)	0	
Canadian Foundation for Healthcare Improvement (formerly the Canadian Health Services Research Foundation)<br><br>http://www.cfhi-fcass.ca/	("knowledge broker" OR "knowledge brokering" OR "knowledge brokerage") AND (health OR medical OR medicine OR nursing OR care)	19	1. http://www.cfhi-fcass.ca/sf-docs/default-source/reports/KnowledgeBrokeringReview-EN.pdf<br>2. http://www.cfhi-fcass.ca/Migrated/PDF/InsightAction/insight_action27_e.pdf<br>3. http://www.cfhi-fcass.ca/Migrated/PDF/InsightAction/insight_action22_e.pdf<br>4. http://www.cfhi-fcass.ca/Migrated/PDF/InsightAction/InsightandAction42f.pdf

SOURCE	SEARCH STRING	SOURCES RETRIEVED	APPLICABLE LINKS
			5. http://www.cfhi-fcass.ca/Migrated/PDF/InsightAction/insight_action23_e.pdf
	"knowledge manager" AND (health OR medical OR medicine OR nursing OR care)	1	none
	"linkage agent" AND (health OR medical OR medicine OR nursing OR care) AND (knowledge-translation OR knowledge-transfer OR knowledge-management OR knowledge-exchange OR knowledge-practice OR knowledge-action OR knowledge-utilization OR knowledge-sharing OR knowledge-mobilization	0	
	"capacity builder" AND (health OR medical OR medicine OR nursing OR care) AND (knowledge-translation OR knowledge-transfer OR knowledge-management OR knowledge-exchange OR knowledge-practice OR knowledge-action OR knowledge-utilization OR knowledge-sharing OR knowledge-mobilization)	0	
Health Evidence<br><br>http://www.healthevidence.org/	("knowledge broker" OR "knowledge brokering" OR "knowledge brokerage") AND (health OR medical OR medicine OR nursing OR care)	1	1. http://www.healthevidence.org/view-article.aspx?a=23166
	"knowledge manager" AND (health OR medical OR medicine OR nursing OR care)	0	
	"linkage agent" AND (health OR medical OR medicine OR nursing OR care) AND (knowledge-translation OR knowledge-transfer OR knowledge-management OR knowledge-exchange OR knowledge-practice OR knowledge-action OR knowledge-utilization OR knowledge-sharing OR knowledge-mobilization	0	
	"capacity builder" AND (health OR medical OR medicine OR nursing OR care) AND (knowledge-translation OR knowledge-transfer OR knowledge-management OR knowledge-exchange OR knowledge-practice OR knowledge-action OR knowledge-	0	

SOURCE	SEARCH STRING	SOURCES RETRIEVED	APPLICABLE LINKS
	utilization OR knowledge-sharing OR knowledge-mobilization)		
Canadian Institutes of Health Research<br><br>http://www.cihr-irsc.gc.ca/	("knowledge broker" OR "knowledge brokering" OR "knowledge brokerage") AND (health OR medical OR medicine OR nursing OR care)	28	1. http://www.cihr-irsc.gc.ca/e/30673.html<br>2. http://www.cihr-irsc.gc.ca/e/45321.html<br>3. http://www.cihr-irsc.gc.ca/e/41594.html<br>4. http://www.cihr-irsc.gc.ca/e/47630.html<br>5. http://www.cihr-irsc.gc.ca/e/documents/icr_quality_cancer_care_workshop_e.pdf<br>6. http://www.cihr-irsc.gc.ca/e/documents/icr_palliative_care_summary_e.pdf<br>7. http://www.cihr-irsc.gc.ca/e/documents/What_a_Difference_Sex_and_Gender_Make-en.pdf<br>8. http://www.cihr-irsc.gc.ca/e/42208.html<br>9. http://www.cihr-irsc.gc.ca/e/documents/ce_handbook_e.pdf
	"knowledge manager" AND (health OR medical OR medicine OR nursing OR care)	0	
	"linkage agent" AND (health OR medical OR medicine OR nursing OR care) AND (knowledge-translation OR knowledge-transfer OR knowledge-management OR knowledge-exchange OR knowledge-practice OR knowledge-action OR knowledge-utilization OR knowledge-sharing OR knowledge-mobilization	0	
	"capacity builder" AND (health OR medical OR medicine OR nursing OR care) AND (knowledge-translation OR knowledge-transfer OR knowledge-management OR knowledge-exchange OR knowledge-practice OR knowledge-action OR knowledge-utilization OR knowledge-sharing OR	0	

SOURCE	SEARCH STRING	SOURCES RETRIEVED	APPLICABLE LINKS
	knowledge-mobilization)		
World Health Organization<br><br>http://www.who.int/	("knowledge broker" OR "knowledge brokering" OR "knowledge brokerage") AND (health OR medical OR medicine OR nursing OR care)	187	**12 articles applicable for screening**<br><br>1. http://www.who.int/bulletin/volumes/84/8/05-028308.pdf<br><br>2. http://www.who.int/workforcealliance/knowledge/publications/alliance/2009KnowledgeStrat.pdf<br><br>3. http://www.who.int/bulletin/volumes/85/5/06-037663/en/<br><br>4. http://applications.emro.who.int/dsaf/dsa999.pdf<br><br>5. http://www.who.int/kms/WHO_EIP_KMS_2006_2.pdf<br><br>6. http://www.who.int/rpc/publications/scaling_up_research.pdf<br><br>7. http://www.who.int/workforcealliance/knowledge/publications/alliance/2009ComStrat_webversion.pdf<br><br>8. http://www.who.int/alliance-hpsr/TR1Healy.pdf<br><br>9. http://www.who.int/workforcealliance/knowledge/public

SOURCE	SEARCH STRING	SOURCES RETRIEVED	APPLICABLE LINKS
			ations/taskforces/refgroup_report2010.pdf<br><br>10. http://www.who.int/bulletin/volumes/87/5/08-058024.pdf<br><br>11. http://whqlibdoc.who.int/publications/2009/9789241598033_eng.pdf<br><br>12. http://www.wpro.who.int/asia_pacific_observatory/about/en/
	"knowledge manager" AND (health OR medical OR medicine OR nursing OR care)	4	0 applicable for screening
	"linkage agent" AND (health OR medical OR medicine OR nursing OR care) AND (knowledge-translation OR knowledge-transfer OR knowledge-management OR knowledge-exchange OR knowledge-practice OR knowledge-action OR knowledge-utilization OR knowledge-sharing OR knowledge-mobilization	0	0 applicable for screening
	"capacity builder" AND (health OR medical OR medicine OR nursing OR care) AND (knowledge-translation OR knowledge-transfer OR knowledge-management OR knowledge-exchange OR knowledge-practice OR knowledge-action OR knowledge-utilization OR knowledge-sharing OR knowledge-mobilization)	2	**1 article applicable for screening**<br>13. http://www.who.int/alliance-hpsr/TR1Healy.pdf
WHO Regional Office for Europe<br>See: BRIDGE (Brokering knowledge and Research Information to support the	("knowledge broker" OR "knowledge brokering" OR "knowledge brokerage") AND (health OR medical OR medicine OR nursing OR care)	10 unique sources (between 61-70 total sources; could not determine specific value. Was	http://www.euro.who.int/en/about-us/partners/observatory/bridge-series<br><br>**5 articles applicable for screening**<br><br>1.

SOURCE	SEARCH STRING	SOURCES RETRIEVED	APPLICABLE LINKS
Development and Governance of health systems in Europe) policy brief and summary series<br><br>http://www.euro.who.int/		not listed on site)	http://www.euro.who.int/__data/assets/pdf_file/0004/195232/Obs-Policy-Brief-17,-How-can-knowledge-brokering-be-advanced-in-a-countrys-health-system.pdf<br><br>2. http://www.euro.who.int/en/about-us/partners/observatory/bridge-series/how-can-knowledge-brokering-be-better-supported-across-european-health-systems<br><br>3. http://www.euro.who.int/__data/assets/pdf_file/0007/195235/Obs-Policy-Summary-9,-Matching-form-to-function.pdf<br><br>4. http://www.euro.who.int/__data/assets/pdf_file/0006/195234/Obs-Policy-Summary-8,-Learning-from-one-another.pdf<br><br>5. http://www.euro.who.int/__data/assets/pdf_file/0005/195233/Obs-Policy-Summary-7,-Communicating-clearly.pdf
	"knowledge manager" AND (health OR medical OR medicine OR nursing OR care)	2	0 applicable for screening
	"linkage agent" AND (health OR medical OR medicine OR nursing OR care) AND (knowledge-translation OR knowledge-transfer OR knowledge-management OR knowledge-exchange OR knowledge-practice OR knowledge-action OR knowledge-utilization OR knowledge-sharing OR	0	0 applicable for screening

SOURCE	SEARCH STRING	SOURCES RETRIEVED	APPLICABLE LINKS
	knowledge-mobilization		
	"capacity builder" AND (health OR medical OR medicine OR nursing OR care) AND (knowledge-translation OR knowledge-transfer OR knowledge-management OR knowledge-exchange OR knowledge-practice OR knowledge-action OR knowledge-utilization OR knowledge-sharing OR knowledge-mobilization)	2	0 applicable for screening
		**340 documents underwent preliminary screening**	**46 documents retained after pre-screening**

November 2014 results provided by Library Services:

1. Kujbida G, Stratton J. Effective knowledge translation tactics for increasing the use of health status and surveillance data. Brampton, ON: Region of Peel; 2014. Available from: http://www.peelregion.ca/health/library/pdf/effective-kt.pdf
2. Bronwynne W, Bergen A. Where do knowledge brokers come from? Presented at 2014 Canadian Knowledge Mobilization Forum. Guelph, ON: University of Guelph; 2014. Available from: http://www.knowledgemobilization.net/archives/7673
3. Child Development & Rehabilitation. Knowledge broker resources [Internet]. Children's & Women's Health Centre of British Columbia; 2014. Available from: http://www.childdevelopment.ca/Evidencecentre/KnowledgeBrokering.aspx
4. Canadian Institutes of Health Research. More about knowledge translation at CIHR [Internet]. CIHR; 2014. Available from: http://www.cihr-irsc.gc.ca/e/39033.html
5. National Collaborating Centre for Healthy Public Policy. Health impact assessment of the Tod Neighbourhood Project in Sainte-Catherine. Report on potential impacts and recommendations. Section 2.1: Knowledge-brokering strategy applied to HIA. Montreal, Quebec: National Collaborating Centre for Healthy Public Policy; 2014. Available from: http://www.ncchpp.ca/docs/2014_EnvBati_BuiltEnv_CLASP_HIA_SteCatherine_EN_Gabarit_Light.pdf
6. Philips D. Mobilize This! Step by step knowledge brokering. ResearchImpact; 2014. Available from: http://researchimpact.ca/step-by-step-knowledge-brokering-le-courtage-des-connaissances-pas-a-pas/
7. Watters N, Kolapak S. Sparking connections: Knowledge Exchange Centre interim report webinar. Mental Health Commission of Canada; 2014. Available from:

http://www.mentalhealthcommission.ca/English/system/files/private/document/MHCC_KEC_SparkingConnections_InterimReportWebinar_Presentation_ENG.pdf

8. Australian Science Communicators. Knowledge brokering in Australia: influencing policy and practice –Eve Merton [Internet]. 2014. Available from: http://2014conf.asc.asn.au/schedule/knowledge-brokering-in-australia-influencing-policy-and-practice-eve-merton/ (includes list of conference papers, a workshop paper, and a diagram on functions of knowledge brokering).
9. Mc Sween E. A knowledge brokering strategy to promote research use in Burkina Faso. 2014. Available from: http://www.equiperenard.ca/knowledge-brokering-strategy-to-promote-research-use-in-burkina-faso/
10. Wang D. Activating brokerage: inter-organizational knowledge transfer through skilled return migration. Columbia University; 2014. Available from: http://isites.harvard.edu/fs/docs/icb.topic1258829.files/WANG-Dan_4-21-14_Activating%20Brokerage%20-%20Inter-organizational%20knowledge%20transfer%20thru%20skilled%20return%20migration_ASQ-2014.pdf
11. Basaza R, Kinengyere A, Sewankambo N. A national framework for sustainability of health knowledge translation initiatives in Uganda. EVIPNet; 2014. Available from: http://www.who.int/evidence/sure/frsustainabilityktuganda.pdf
12. East African Community Health Sector. Knowledge brokers link health research, policy and practice [Internet]. East African Community; 2014. Available from: http://www.eac.int/health/index.php?option=com_content&view=article&id=98&Itemid=147
13. Lemire N, Souffez K, Laurendeau MC. Facilitating a knowledge translation process: knowledge review and facilitation tool. Gouvernement du Québec; 2013. Available from: http://www.inspq.qc.ca/pdf/publications/1628_FaciliKnowledgeTransProcess.pdf
14. Malinovskyte M, Mothe C, Ruling CC. Knowledge brokerage: towards an integrative conceptual framework. AIMS Association Internationale de Management Strategique. n.d. Available from: http://www.strategie-aims.com/events/conferences/24-xxiiieme-conference-de-l-aims/communications/3166-knowledge-brokerage-towards-an-integrative-conceptual-framework/download
15. SATNET Asia. SATNET shares its experience as a knowledge broker with the Regional Open Innovation Forum in Malaysia. SATNET; 2012. Available from: http://www.satnetasia.org/news03-11-2014b.html
