# Supplementary material for: Exploring the function and effectiveness of knowledge brokers as facilitators of knowledge translation in health-related settings: a systematic review and thematic analysis
Source: Implement Sci. 2015 Nov 20;10:162. doi: 10.1186/s13012-015-0351-9 (PMC4653833; doi:10.1186/s13012-015-0351-9)
Supplement: Additional file 3: — Summary of KB characteristics. (http://www.implementationscience.com/imedia/5868567011931448/supp3.pdf). (PDF 237 kb) [file 13012_2015_351_MOESM3_ESM.pdf]

Additional File 3. Summary of characteristics from studies investigating the use of knowledge brokers in health-related settings.

Study	First author (year) country of first author	Study design	KT setting	Purpose of KT initiative	Duration of KB initiative[1]	KB level of experience[2]	KB Position status	Approach and number of KBs	Internal or external KB role
1	Chew et al. [47] (2013) England	Qualitative; grounded theory	Academic; mixed healthcare settings	To improve patient outcomes by adapting research to fit the needs of practice and increasing stakeholder participation in research	Unspecified	Novice	Full-time	Multiple independent KBs (n = 7)	Internal
2	Conklin et al. [15] (2013) Canada	Mixed methods; multiple case study	Community of practice; government; academic	To connect stakeholders to improve knowledge exchange to enhance seniors' health	5 years	Unspecified	Unspecified	Multiple independent KBs (n = 6 in year 4, n = 5 in year 5; years 1-3 = unspecified)	Internal
3	Hoens et al. [41] (2013) Canada	Qualitative; case study	Clinical	To support evidence informed practice in respiratory care	4 years	Unspecified	Full-time	Independent KB (n = 1)	Unspecified
4	Waqa et al. [35][1] (2013) Fiji	Mixed methods; case study	Academic; government; non-government organizations	To advance evidence-informed policy development to improve eating and physical activity environments	12-18 months	Unspecified	Full-time and part-time	KB team (n = 1 KB); Other members: research fellow, academic consultant, policy advisors	External
	Waqa et al. [34][1] (2013) Fiji	Qualitative; case study	Academic; government; non-government organizations	To advance evidence-informed policy development to improve	3 years	Unspecified	Full-time and part-time	KB team (n = 1 KB); Other members: research fellow, academic	External

				eating and physical activity environments				consultant, policy advisors	
5	Waring et al. [49] (2013) England	Qualitative; ethnography	Clinical	To facilitate inter-departmental knowledge exchange in hospital settings	3 years	Unspecified	Part-time	Multiple independent KBs (n = 19)	Internal
6	Frank et al. [34] (2012) Scotland	Qualitative; case study	Academic; public health	To foster collaborative research among stakeholders to impact policies, programs and practice in public health	3 years	Unspecified	Unspecified	KB organization (n = unspecified)	External
7	Ward et al. [43] (2012) England	Mixed methods; multiple case study	Primary care	To assess knowledge exchange within a large mental health organization	10-15 months	Unspecified	Unspecified	Independent KB (n = 1)	External
8	Cameron et al. [44] (2011)[2] Canada	Qualitative; exploratory	Mixed healthcare settings	To facilitate use of evidence-based practice among physiotherapists working with children with cerebral palsy	6 months	Unspecified	Unspecified	Multiple independent KBs (n = 28)	Internal
	Rivard et al. [39] (2010) Canada	Mixed method; descriptive	Mixed pediatric healthcare facilities (hospitals, rehabilitation centers)	To facilitate the uptake of measurement tools by physiotherapists in pediatric settings	6 months	Novice	Part-time	Multiple independent KBs (n = 24)	Internal
	Russell et al. [52] (2010) Canada	Mixed methods; longitudinal	Mixed pediatric healthcare facilities (hospitals, rehabilitation centers)	To facilitate the uptake of measurement tools by physiotherapists in pediatric settings	18 months	Novice	Part-time	Multiple independent KBs (n = 24)	Internal

9	Campbell et al. [36] (2011) Australia	Qualitative; multiple case study	Government; Academic	To provide policy makers with rapid, structured reviews of evidence	3 years	Experienced	Part-time	KB organization; multiple independent KBs (n = unspecified)	External
10	Gerrish et al. [45] (2011) England	Qualitative; case study	Clinical	To understand how advanced practice nurses use and share evidence in practice	Unspecified	Unspecified	Unspecified	Multiple independent KBs (n = 23)	Internal
11	Urquhart et al. [29] (2011) Canada	Qualitative; case study	Academic; clinical; government	To improve access to quality colorectal cancer care	5 years	Novice	Full time	Independent KB (n = 1)	Internal
12	Jansson et al. [31] (2010) Canada	Qualitative; reflection	Non-government organizations	To enhance collaborative partnership among stakeholders working with adult persons working in sex industry	Unspecified	Unspecified	Unspecified	Multiple independent KBs (n = 2)	Internal
13	Kimble et al. [40] (2010) France	Qualitative; multiple case study	Clinical	To facilitate knowledge exchange between members of a neurological/ cognitive condition network	Unspecified	Unspecified	Unspecified	Independent KB (n = 2)[3]	Internal
14	Dobbins et al. [46] (2009) Canada	Randomized controlled trial	Public health	To facilitate incorporation of research evidence into public health policies and programs	2 years	Novice[4]	Full-time and part-time staff	Multiple independent KBs (n = 2)	External
	Dobbins et al. [21] (2009) Canada	Qualitative; reflection	Public health	To facilitate incorporation of research evidence into public health policies and programs	2 years	Novice[4]	Full-time	Independent KB (n = 1)	External
	Robeson et al. [38] (2008) Canada	Qualitative; reflection	Public health	To facilitate incorporation of research evidence into	2 years	Novice[4]	Full-time	Independent KB (n = 1)	External

				public health policies and programs					
	Traynor et al. [48] (2014) Canada[5]	Secondary analysis of qualitative data; case study	Public health	To facilitate incorporation of research evidence into public health policies and programs	Unspecified	Novice[4]	Unspecified	Unspecified	Unspecified
15	Richards et al. [30] (2009) England	N/A; editorial	Government; public health	To facilitate collaboration between politicians and academics to enhance public health	10 years	Unspecified	Unspecified	KB organization; (n = unspecified)	External
16	Healy et al. [28] (2007) Australia	N/A; grey literature report	Academia; government	To connect researchers, policy makers and health services to maximize the value of health research	Unspecified	Unspecified	Unspecified	KB organization; multiple independent KBs (n = unspecified)	External
17	Healy et al. [28] (2007) Australia	N/A; grey literature report	Academia; government	To support well-informed health policy across the WHO European region	Unspecified	Unspecified	Unspecified	KB organization; (n = 20)	External
18	van Kammen et al. [17] (2006) Netherlands	Qualitative; multiple case study	Academic; government	<u>Netherlands case</u>: To support evidence-based and context-informed decision making; <u>East Africa case</u>: To improve health and equity through evidence informed health policies and practices	Unspecified	Unspecified	Unspecified	KB organization; (n = 2; individual membership unspecified)	External
	van Kammen et al. [51] (2006) Netherlands	Qualitative; case study	Academic; government; health insurance board	To facilitate evidence-informed decision making related to subfertility care and reimbursements	Unspecified	Unspecified	Unspecified	KB organization; (n = unspecified)	External

19	Lyons et al. [42] (2006) Canada	N/A; grey literature report	Academic; government; non-government organizations	To increase decision maker uptake of best practices in integrated stroke care	3 years	Unspecified	Unspecified	Multiple independent KBs (n = 4)	External
20	Stevens et al. [37] (2005) England	Mixed methods; case study	Government; non-government organizations	To promote the use of good quality research evidence in social care practice with children	Unspecified	Unspecified	Unspecified	Independent KB (n = 1)	External
21	Yost et al. [50] (2014) Canada	Qualitative; multiple case study	Public health	To enhance capacity for and facilitate organizational contexts conducive to evidence informed decision making	Case A: 17 months; Case B: 22 months; Case C: 20 months	Unspecified	Part-time	Multiple independent KBs (n = 3)	External
	Traynor et al. [48] (2014) Canada[4]	Secondary analysis of qualitative data; case study	Public health	To enhance capacity for and facilitate organizational contexts conducive to evidence informed decision making	22 months	Unspecified	Unspecified	Unspecified	Unspecified
22	Donnelly et al. [35] (2014) Canada	Qualitative; case study	Primary care	To facilitate the application of evaluation knowledge into practice at a memory clinic.	8 months	Unspecified	Unspecified	Independent KB (n = 1)	Unspecified

[1]Some studies (e.g., study 8) include articles that feature findings at differing stages of completion.

[2]KBs with no reported prior knowledge brokering experience were deemed “novice”; KBs with any reported brokering experience were deemed “experienced”.

[3]Note: Two KBs were involved in the study; however, the positions were held consecutively, not concurrently.

[4]While the KBs were masters-prepared and experienced in the public health field, they did not have explicit experience working as a knowledge broker

[5]Traynor et al. reflects on studies 14 and 21

KT=knowledge translation; KB=knowledge broker; N/A=not applicable
