# Supplementary material for: Exploring the function and effectiveness of knowledge brokers as facilitators of knowledge translation in health-related settings: a systematic review and thematic analysis
Source: Implement Sci. 2015 Nov 20;10:162. doi: 10.1186/s13012-015-0351-9 (PMC4653833; doi:10.1186/s13012-015-0351-9)
Supplement: Additional file 4: — Summary of MetaQAT appraisals. (http://www.implementationscience.com/imedia/1334272237170299/supp4.pdf). (PDF 352 kb) [file 13012_2015_351_MOESM4_ESM.pdf]

Additional File 4. Summary of MetaQAT appraisals from studies investigating the use of knowledge brokers in health-related settings

MetaQAT Criteria		MetaQAT Assessment Questions
Relevancy	Does the study address a topic(s) relevant to the issue under investigation?	**[1]** Was study justification clearly stated? **[2]** Do the results of the study apply to the issue under consideration? **[3]** How similar or different is study population/setting to yours? Is a difference likely to matter? **[4]** Is the research design appropriate for methodology?
Reliability	Is the study presented clearly?	**[1]** Is rationale for study clearly stated? Does study focus on a clearly defined issue? **[2]** Is conduct of study clearly described and easy to follow? **[3]** Can you identify the research design? **[4]** Are all results included? **[5]** Are findings presented and discussed within the appropriate context? **[6]** Is there a conflict of interest statement? **[7]** Can the study be reproduced with the information provided?
	Are the research methodology and results clearly described?	**[1]** Does methodology describe population studied, intervention given, and outcomes? **[2]** Are all data sources clearly identified? **[3]** Are inclusion/exclusion criteria defined? **[4]** Are statistical/analytical methods described? **[5]** Are data tables consistent with results section? **[6]** Could methods be reproduced based on information provided?
	Are ethics procedures described?	**[1]** Was appropriate informed consent obtained? **[2]** Was the study approved by an ethics review board?
Validity	Is the study methodology appropriate for the scope of research?	**[1]** Is research question congruent with study design? **[2]** Does the methodology match the theory or conceptual model? **[3]** Are important theoretical factors accounted for in analysis? **[4]** Are statistical/analytical methods appropriate for the design and/or the question? **[5]** Are important theoretical factors accounted for in analysis?
	Is the research methodology free from bias?	**[1]** Were there major sources of bias related to: study design? Participants' inclusion or exclusion? Measurement of exposure/outcome or important confounders or predictors? Data sources? Quality assessed? Selection of studies? **[2]** Are all comprehensive factors included in the research? **[3]** Are results consistent within the study? **[4]** Can chance findings be ruled out? **[5]** Were analyses carried out appropriately?
	Are the authors' conclusions explicit and transparent?	**[1]** Are the results conclusive? **[2]** Are conclusions clearly derived from the results (i.e. transparent)? **[3]** Are potential discrepancies discussed?
	Can I be confident about findings?	**[1]** Are there any major methodological flaws that limit the validity of findings? **[2]** Are the study's results similar to those of the existing body of literature? If not, are the reasons for the difference clearly explained?
Applicability	Can the results be applied within a health-related scope?	**[1]** Can the study results be interpreted and analyzed within the context of health? **[2]** Are there other important health outcomes to be considered that were not included? **[3]** Can the results be applied to (public) health practice, based on the validity of the article and its relevance? **[4]** Are harms and benefits discussed? **[5]** Were the relevant stakeholders consulted?
**Study 4: Waqa, et al. [34,35]**		
Relevancy	Does the study address a topic(s) relevant to the issue under investigation?	**[1,2]** Study addresses how KBs operate in practice **[3]** Differences related to geographic and policy-related generalizability **[4]** No methodological exclusion criteria
Reliability	Is the study presented clearly?	**[1]** Rationale clearly stated **[2]** Study described well however, another paper referenced for full protocol/ measurement tools; limits clarity **[3]** Research design identifiable **[4]** Not all results included (no data regarding participants' knowledge of evidence-informed policymaking prior to the training workshops presented, despite

		study protocol which indicated that these data would be collected) **[5]** Findings discussed in appropriate context **[6]** Conflict of interest statement present **[7]** Study could be reproduced
	Are the research methodology and results clearly described?	**[1]** Inadequate description of population studied (KB team: no description of prior KB experience, training or KBs' areas of expertise; Organizational participants: no data for years of employment, previous experience with policy briefs); intervention (inadequate description of workshops and support preparing policy briefs); and outcomes (no quality appraisal of policy briefs, no policy changes) **[2]** Not all data sources clearly identified (e.g., quality assessment of policy briefs not identified) **[3]** Inclusion criteria defined **[4]** Statistical/analytical methods described clearly **[5]** Data tables inconsistent with results section (analysis of organizational capacity data presented in Table 2 of [35] citation, but results discussed in [34] citation) **[6]** Methods could not be reproduced in entirety (analysis of process diaries not described)
	Are ethics procedures described?	**[1]**Consent obtained **[2]** Ethical approval granted
Validity	Is the study methodology appropriate for the scope of research?	**[1]** Research question not congruent with design; but design was congruent with objectives **[2]** Methodology matches theory **[3, 4]** Analytical methods not appropriate for design/research question. (Policy brief completion deemed "evidence of skill development" but no quality assessment of policy briefs; no evidence to suggest policy briefs resulted in changes to policies or practices.)
	Is the research methodology free from bias?	**[1]** Potential bias: participants' inclusion or exclusion (relationship between participant skill level/role and quantity/quality of policy briefs not assessed. No demographic data for those who completed the program (n=27) in contrast to those who did not (n=22)); measurement of confounders (participants interviewed by KB team member who may have trained them); quality assessed (quality of policy briefs not assessed) **[2]** Some factors critical to interpretation were excluded (KBs level of experience, participant's previous experience with policy brief creation) **[3]** Results consistent within the study **[4]** Chance cannot be ruled out (no confidence intervals; no site-specific analyses or comparisons) **[5]** Appropriate analyses in both studies; however no discussion of inter-rater reliability or member-checking
	Are the authors' conclusions explicit and transparent?	**[1]** Results not conclusive: "Policy brief completion" used as indicator of skill development, but no quality appraisal of policy briefs **[2]** Conclusions did not align with central research objective (i.e., to determine if a KB approach could advance evidence-informed *policy development*) - there was a disconnect between the development of policy *briefs* versus development of actual *policies* **[3]** Potential discrepancies not discussed between n=27 participants who completed the program and n=20 policy briefs submitted
	Can I be confident about findings?	**[1]** Validity of findings limited by methodological concerns: outcomes not clearly described; no baseline assessment of evidence use in decision making/policy brief creation **[2]** Study results were positive in citing KB team as an effective strategy for KT. Reasons for discrepancies between study findings and existing literature discussed
Applicability	Can the results be applied within a health-related scope?	**[1]** Study results could be interpreted and analyzed within the context of health if appropriate methodological considerations addressed (e.g., potential biases, nature of results reported) **[2]** Other important outcomes not included and should be considered (e.g., policy development stemming from policy brief creation; quality of policy briefs) **[3]** Data necessary for discerning applicability of findings not presented (no quality appraisal of

		policy briefs; no description of how KB intervention related to changes in policy/knowledge) **[4]** Key harms and benefits discussed **[5]** Relevant stakeholders were consulted, however, incomplete description of KB and participants who completed full intervention (i.e., insufficient understanding of context and n=27 participants who completed full intervention – n=20 of whom presumably submitted policy briefs - and how these individuals differ from the n=22 who dropped out)
**Study 7: Ward et al. [43]**		
Relevancy	Does the study address a topic(s) relevant to the issue under investigation?	**[1,2]** Study addresses how KBs operate in practice **[3]** Potential differences related to geographic generalizability **[4]** No methodological exclusion criteria
Reliability	Is the study presented clearly?	**[1]** Rationale clearly stated **[2]** Study described well when protocol/supplementary files are considered **[3]** Research design identifiable **[4]** Not all results included (i.e., limited information on KB activities, training, experience) **[5]** Findings discussed in appropriate context; however, important outcome data presented only in supplemental file **[6]** Conflict of interest statement present **[7]** Study could be reproduced
	Are the research methodology and results clearly described?	**[1]** Methodology clearly described <u>population studied</u> and <u>outcomes</u>. Did not clearly describe <u>intervention</u> (i.e., KB activities) **[2]** Data sources identified (e.g., field notes from KB; participant interviews; document reviews); however, no search terms included for the literature search **[3]** Inclusion/exclusion criteria not defined **[4]** Analytical methods described (realist, 'process tracing' approach) **[5]** Data tables consistent with results section **[6]** Methods not described clearly enough to reproduce study (no information on KB training, experience, or KB activities)
	Are ethics procedures described?	**[1]** Consent process not described **[2]** Ethical approval granted
Validity	Is the study methodology appropriate for the scope of research?	**[1]** Research question congruent with design **[2]** Methodology matched theory/conceptual model; however, the effectiveness of KBs as a means to facilitate knowledge exchange was not clearly connected to conceptual model **[3]** Analytical methods were appropriate for design and research question ('process tracing', thematic analysis) **[4]** Important theoretical factors not accounted for in analysis (e.g., how effective KBs were in their settings and how this impacted knowledge exchange)
	Is the research methodology free from bias?	**[1]** Potential bias: <u>participants</u> (no information on KB training/experience; limited information on service delivery participants), <u>setting</u> (did not provide sufficient connections to policymakers' concerns), <u>methods</u> (limited information on KB activities; no statement of author's personal potential biases), and <u>measurement of outcome/confounders</u> (no statement on the quality or use of evidence in the policy briefs developed by the participants to verify this assertion) **[2]** Some factors critical to interpretation not described (e.g., no information on length of time KB spent with teams or the nature or quantity of support provided by KB; did not evaluate participants' perceptions of valid knowledge sources prior to the KB intervention; did not evaluate changes in participants' knowledge after working with the KB) **[3]** Results of KB success were not consistent (Team 1 achieved some goals with uncertain sustainability; Team 2 achieved goals; Team 3 did not achieve goals) *from suppl. file **[4]** Site-specific comparisons reduced possibility of chance findings; however, effectiveness of KBs may

		differ based on mixed success rate of teams **[5]** Appropriate analyses; but no member-checking or interpretation verification
	Are the authors' conclusions explicit and transparent?	**[1]** Results related to success of knowledge exchange sites (in suppl. file) were not presented clearly **[2]** Conclusions clearly derived from results **[3]** Potential discrepancies not discussed related to effectiveness of KBs to support/facilitate knowledge exchange
	Can I be confident about findings?	**[1]** Validity of findings limited by methodological concerns: insufficient description of KB training/experience and service delivery participants; limited information on KB activities in each setting **[2]** Results were mixed relative to the KBs' ability to facilitate knowledge exchange at each site *from suppl. file
Applicability	Can the results be applied within a health-related scope?	**[1]** Study results could be interpreted and analyzed within the context of health if appropriate methodological considerations considered **[2]** Other important outcomes not included and should be considered (e.g., policy implications) **[3]** Generally, the results (i.e., framework) could be applied in other health settings, however, because very few details provided about the participants and the KB, it is difficult to generalize the findings **[5]** Not all relevant stakeholders consulted (e.g., no member-checking to verify theoretical assumptions; no clear consideration of policy implications)
**Study 8: Russell et al. [52]; Cameron et al. [44]; Rivard et al. [39]**		
Relevancy	Does the study address a topic(s) relevant to the issue under investigation?	**[1,2]** Study addresses how KBs operate in practice **[3]** No significant issues with generalizability **[4]** No methodological exclusion criteria
Reliability	Is the study presented clearly?	**[1]** Rationale clearly stated **[2]** Study described well and easy to follow **[3]** Research design identifiable **[4]** All results are included (when all articles considered collectively) **[5]** Findings presented in appropriate context **[6]** Conflict of interest statement present **[7]** Study could be reproduced
	Are the research methodology and results clearly described?	**[1]** Methodology describes the population studied, (physiotherapist KBs) intervention (KB network, broker to the KBs, online support, interactive workshop), and outcomes (change in self-reported knowledge and use of measurement tools; KB process evaluation) **[2]** All sources of information identified **[3]** Inclusion/exclusion criteria defined **[4]** Statistical and analytical methods described **[5]** Data tables consistent with results section **[6]** Methods could be reproduced
	Are ethics procedures described?	**[1]** Consent obtained **[2]** Ethical approval granted
Validity	Is the study methodology appropriate for the scope of research?	**[1]** Research question congruent with design **[2]** Methodology matched conceptual model **[3]** Statistical and analytical methods appropriate for design and research question **[4]** Important theoretical factors accounted for in the analysis (e.g., clear connections between KB intervention and KtoA theoretical framework)
	Is the research methodology free from bias?	**[1]** No major sources of bias identified **[2]** Comprehensive factors critical to interpretation included in research **[3]** Results consistent within the study **[4]** Chance findings can be ruled out (i.e., confidence interval data; region-specific analyses and comparisons) **[5]** Analyses carried out appropriately
	Are the authors' conclusions explicit and transparent?	**[1]** Results were conclusive **[2]** Conclusions clearly derived from results **[3]** Potential discrepancies were discussed relative to geographic differences, research design, and sample size

	Can I be confident about findings?	**[1]** No major methodological flaws that limit validity of findings **[2]** Where outcome data conflicted with existing literature, authors provided sound reasons for discrepancies in findings
Applicability	Can the results be applied within a health-related scope?	**[1]** Results could be interpreted/analyzed within health context **[2]** Key important outcomes were included and evaluated (change in knowledge and practice) **[3]** Results could be applied to health-related practice based on the validity and relevance of the article **[5]** Relevant stakeholders consulted
**Study 9: Campbell et al. [36]**		
Relevancy	Does the study address a topic(s) relevant to the issue under investigation?	**[1,2]** Study addresses how KBs operate in practice **[3]** Potential issues related to generalizability **[4]** No methodological exclusion criteria
Reliability	Is the study presented clearly?	**[1]** Rationale clearly stated **[2]** Study not described well **[3]** Research design not identifiable **[4]** Not all results included (no KB feedback; Evidence Check limitations; contextual factors) **[5]** Findings discussed in appropriate context; but no KB perspectives included **[6]** No conflict of interest statement provided **[7]** Study could not be reproduced
	Are the research methodology and results clearly described?	**[1]** Methodology did not adequately describe: <u>population</u> (no data on participants' professional background or research/policy experience), <u>intervention</u> (no review appraisal), <u>outcomes</u> (no methods for evaluating effectiveness of KBs) **[2]** Data sources not clearly described (no descriptive data on participants; interview content not described) **[3]** No inclusion/exclusion criteria **[4]** No analytical methods described **[5]** Data tables consistent with results section **[6]** Methods could not be reproduced based on information provided
	Are ethics procedures described?	**[1]** Consent process not described **[2]** Ethical approval not described
Validity	Is the study methodology appropriate for the scope of research?	**[1]** No research design/question specified **[2]** Evidence Check process appeared to match underlying theory; research methodology not clearly described **[3]** Analytical methods not described **[4]** Important theoretical factors not accounted for in the analysis (experience of KB, political climate)
	Is the research methodology free from bias?	**[1]** Potential bias: <u>design</u> (no design identified; did not identify how participants were selected or recruited); <u>participants</u> (no inclusion/exclusion criteria); <u>outcome measurement</u> (no KB perspectives included); <u>quality assessed</u> (no quality appraisal conducted) **[2]** Factors critical to interpretation not included (KB perspectives, impact of political climate) **[3]** Results consistent within the study **[4]** Chance findings cannot be ruled out (no site comparisons) **[5]** Unable to assess appropriateness of analyses; not described sufficiently
	Are the authors' conclusions explicit and transparent?	**[1]** Results not conclusive: did not describe challenges with process; did not include KBs perspectives; did not describe how reviews were used by decision makers; no comment on the effectiveness of KBs **[2]** Conclusions not clearly derived from results (no description of direct impacts on policy or practice) **[3]** Potential discrepancies not discussed
	Can I be confident about findings?	**[1]** Validity of findings limited by methodological concerns: no research design identified; no participant selection/recruitment; no inclusion/exclusion criteria; no KB perspectives; no assessment of effectiveness **[2]** Study results cited KBs as an effective strategy for KT in health policy contexts
Applicability	Can the results be applied within a health-related scope?	**[1]** Results could likely not be interpreted and analyzed within the context of health policy; data necessary for discerning reliability and generalizability not presented **[2]** Other important outcomes not included and should be

		considered (e.g., KB perspectives, effectiveness of KBs, political climate) **[3]** Results could not be applied to health policy initiatives based on the validity of this article **[5]** Not all relevant stakeholders were consulted (e.g., KBs)
**Study 14: Dobbins, et al. [21,46]; Traynor et al. [48]; Robeson et al [38]**		
Relevancy	Does the study address a topic(s) relevant to the issue under investigation?	**[1,2]** Study addresses how KBs operate in practice **[3]** No significant issues with generalizability **[4]** No methodological exclusion criteria
Reliability	Is the study presented clearly?	**[1]** Rationale clearly stated **[2]** Study described well **[3]** Research design identifiable (RCT) **[4]** All results included **[5]** Findings presented and discussed in appropriate context **[6]** Conflict of interest statement present **[7]** Study could be reproduced
	Are the research methodology and results clearly described?	**[1]** Methodology described population studied, intervention given, and outcomes **[2]** Data sources clearly identified **[3]** Inclusion/exclusion criteria defined **[4]** Analytical methods described **[5]** Data tables consistent with results section **[6]** Methods could be reproduced based on information provided
	Are ethics procedures described?	**[1]** Consent obtained **[2]** Ethical approval granted
Validity	Is the study methodology appropriate for the scope of research?	**[1]** Research question congruent with design **[2]** Methodology matched conceptual model **[3]** Statistical and analytical methods appropriate for RCT design and research question **[4]** Important theoretical factors accounted for in the analysis (e.g., multiple measures of evidence-informed decision making)
	Is the research methodology free from bias?	**[1]** No major biases identified **[2]** Factors critical to interpretation were included **[3]** Results consistent within the study **[4]** Chance findings cannot be ruled out (two KBs used independently, but cannot account for individual-level factors that may have influenced the effectiveness of the KB, e.g., soft skills) **[5]** Analyses carried out appropriately
	Are the authors' conclusions explicit and transparent?	**[1]** Results conclusive **[2]** Conclusions clearly derived from results **[3]** Potential discrepancies discussed related to organizational value of research evidence and passive vs. active methods of KTE
	Can I be confident about findings?	**[1]** No major methodological flaws that limit the validity of findings **[2]** Study results did not find KBs to be an effective strategy for KT in public health settings; results situated in current literature
Applicability	Can the results be applied within a health-related scope?	**[1]** Results could be interpreted/analyzed within health context **[2]** Key important outcomes were included and evaluated (change in policy/practice) **[3]** Results could be applied to health-related practice, based on the validity of the article and its relevance **[5]** Relevant stakeholders consulted
**Study 18: van Kammen et al. [17,51]**		
Relevancy	Does the study address a topic(s) relevant to the issue under investigation?	**[1,2]** Study addresses how KBs operate in practice **[3]** Potential issues related to geographic generalizability **[4]** No methodological exclusion criteria
Reliability	Is the study presented clearly?	**[1]** Rationale clearly stated **[2]** Study not described well nor easy to follow ("step 1" describes evidence synthesis with no description of study selection or appraisal) **[3]** Research design not evident **[4]** Not all results included (e.g., synthesis) **[5]** Policy-specific findings discussed in appropriate context for ZonMw initiative **[6]** Conflict of interest statement present **[7]** Study could not be reproduced

	Are the research methodology and results clearly described?	**[1]** Methodology did not adequately describe: population (no data on experience, credentials, organizational role), intervention (insufficient detail to reproduce). Methodology adequately described: outcomes for ZonMw KB initiative (infertility policy changes) but not REACH initiative **[2]** Sources of information not clearly identified **[3]** Exclusion of pharmaceutical industry individuals **[4]** No analytical methods described **[6]** Methods could not be reproduced
	Are ethics procedures described?	**[1]** Consent process not described **[2]** Ethical approval not described
Validity	Is the study methodology appropriate for the scope of research?	**[1]** No research design/question specified **[2]** No guiding theory; cannot assess alignment with methodology **[3]** Analysis not described; cannot assess appropriateness of methodology **[4]** No guiding theory/conceptual model; cannot assess alignment with analysis
	Is the research methodology free from bias?	**[1]** Potential bias: study participants (pharmaceutical reps excluded; other participants not described clearly); data sources (not described clearly, no specific data sources informing policy decisions referenced) **[2]** Factors critical to interpretation not included (ZonMw activities not described clearly) **[3]** Results consistent within the study **[4]** Chance findings cannot be ruled out (no site-specific analyses or comparisons) **[5]** Analyses not described adequately enough to assess potential sources of bias
	Are the authors' conclusions explicit and transparent?	**[1]** Results conclusive for ZonMw initiative **[2]** Conclusions derived from results **[3]** Potential discrepancies discussed (political cause of failure to adopt 2nd recommendation)
	Can I be confident about findings?	**[1]** Validity of findings limited by methodological concerns: (no conceptual model, research design and analysis not described) **[2]** Results were positive in citing KBs as an effective strategy for KT, but acknowledged contextual limitations and considerations that may limit the effectiveness of KBs (e.g., political will)
Applicability	Can the results be applied within a health-related scope?	**[1]** Results cannot be interpreted/analyzed within the context of health based on the validity of this article **[2]** Other important outcomes not included and should be considered (e.g., follow-up actions stemming from policy decision, KB approach) **[3]** Results cannot be applied to other health-related settings based on the validity of this article (i.e., the contribution of the KB as a facilitator of this change cannot be determined based on the information contained in this report) **[5]** Relevant stakeholders were consulted.
**Study 19: Lyons et al. [42]**		
Relevancy	Does the study address a topic(s) relevant to the issue under investigation?	**[1,2]** Study addresses how KBs operate in practice **[3]** No significant issues with generalizability **[4]** No methodological exclusion criteria
Reliability	Is the study presented clearly?	**[1]** Rationale clearly stated **[2]** Study not described well (no methods/outcome evaluation; NB: study in progress at time of report) **[3]** Research design not identifiable; could not be reproduced **[4]** Not all results included **[5]** Findings presented in appropriate context **[6]** No conflict of interest statement **[7]** Study could not be reproduced with information provided
	Are the research methodology and results clearly described?	**[1]** Methodology did not adequately describe: population studied (did not indicate who was interviewed, or when), intervention (time/intensity of KB initiative not described; no site comparison), or outcomes (study in progress) **[2]** Data sources not clearly identified **[3]** Inclusion/ exclusion criteria not defined **[4]** Analytic methods not described **[6]** Methods could not be reproduced

	Are ethics procedures described?	**[1]** Consent process not described **[2]** Ethical approval not described
Validity	Is the study methodology appropriate for the scope of research?	**[1]** Research objective congruent with design **[2]** No theory/conceptual model so cannot assess alignment with methodology **[3]** Cannot assess appropriateness of methodology (analysis not described) **[4]** No guiding theory/conceptual model so cannot assess alignment with analysis
	Is the research methodology free from bias?	**[1]** Potential bias: design (no comparison between n=6 participant organizations); participants (no demographic data on participants or KBs; no exclusion criteria); measurement of outcome/confounders (no pre/post-KB intervention comparisons; study in progress at time of report); quality assessed (no objective quality appraisal) **[2]** Factors critical to interpretation not included (comparisons between sites and pre/post-KB intervention) **[3]** Results not consistent within the study **[4]** Chance findings cannot be ruled out (no site comparisons) **[5]** Cannot assess appropriateness of analysis (not described)
	Are the authors' conclusions explicit and transparent?	**[1]** Results inconclusive; study in progress **[2]** Conclusions not clearly derived from results (no discussion of analytic technique used to discern key findings) **[3]** Potential discrepancies discussed related to timing of partnerships, project goal negotiation, provincial priorities related to stroke, divergent mandates
	Can I be confident about findings?	**[1]** Validity of findings limited by methodological concerns: no guiding conceptual model; no comparisons between sites and pre/post-KB intervention; unidentifiable study design; participants; data sources; outcome measurement **[2]** Results were positive in citing KBs as an effective strategy for KT, but noted contextual factors that may limit effectiveness of KBs (e.g., organizational priorities, funding, and timing)
Applicability	Can the results be applied within a health-related scope?	**[1]** Results should not be interpreted within health context until study is complete and full appraisal of methods and results can be performed **[2]** Other important outcomes were not included and should be considered (pre- and post-KB comparison of team communication) **[3]** Data necessary for discerning applicability of findings not presented **[4]** Key harms and benefits discussed (organizational priorities, funding, and timing) **[5]** Relevant stakeholders were consulted
**Study 21: Yost et al. [50]; Traynor et al. [48]**		
Relevancy	Does the study address a topic(s) relevant to the issue under investigation?	**[1,2]** Study addresses how KBs operate in practice **[3]** No significant issues with generalizability **[4]** No methodological exclusion criteria
Reliability	Is the study presented clearly?	**[1]** Rationale clearly stated **[2]** Study described well and easy to follow **[3]** Research design identifiable **[4]** All results not included (quantitative results to be included in upcoming paper) **[6]** Conflict of interest statement present **[7]** Study could not be reproduced (no detailed description of measurement of change in knowledge, skill, practice)
	Are the research methodology and results clearly described?	**[1]** Methodology describes population studied, and intervention given; but insufficient description of quantitative measurement of change in knowledge, skill, practice **[2]** All data sources not identified (measurement of change in knowledge, skill, practice) **[3]** Inclusion and exclusion criteria defined **[4]** Analytical methods not described in entirety (quantitative measurement of change in knowledge, skill, practice) **[5]** Results in tables not consistent with results section **[6]** Methods could not be reproduced

	Are ethics procedures described?	**[1]** Consent obtained **[2]** Ethical approval granted
Validity	Is the study methodology appropriate for the scope of research?	**[1]** Research question congruent with design **[2]** Methodology matches conceptual model **[3]** Analytical methods are appropriate **[4]** Important theoretical factors are accounted for in the analysis (e.g., McKinsy 7-S model informed coding structure)
	Is the research methodology free from bias?	**[1]** Potential bias: study design (no comparison of tool use at sites without a KB); sampling (possible lack of data saturation); outcome measurement (KB who completed intervention conducted interviews) **[2]** Not all critical factors included (contextual factors not fully described) **[3]** Results consistent within the study **[4]** Chance findings cannot be ruled out (no comparisons with sites that did not have a KB) **[5]** Analyses carried out appropriately
	Are the authors' conclusions explicit and transparent?	**[1]** Results conclusive **[2]**Conclusions derived from results **[3]** Potential discrepancies not discussed related to the differences between cases in their use of the tools
	Can I be confident about findings?	**[1]** Validity of findings limited by methodological concerns: inadequate description of quantitative measurement of change in knowledge, skill, practice; KB who completed intervention conducted interviews; contextual factors not fully described **[2]** Results similar to those of the existing body of literature
Applicability	Can the results be applied within a health-related scope?	**[1]** Results could be interpreted within health context if appropriate methodological considerations accounted for (case study design limits transferability, potential bias in measurement of outcomes) **[2]** Other important outcomes not included (baseline measures; use of tools without KB) **[3]** Results could be applied to health-related practice **[4]** Key harms and benefits not discussed **[5]** Relevant stakeholders were consulted
